# SHIP1 Is Present but Strongly Downregulated in T-ALL, and after Restoration Suppresses Leukemia Growth in a T-ALL Xenotransplantation Mouse Model

**DOI:** 10.3390/cells12131798

**Published:** 2023-07-06

**Authors:** Patrick Ehm, Ruth Rietow, Wiebke Wegner, Lara Bußmann, Malte Kriegs, Kevin Dierck, Stefan Horn, Thomas Streichert, Martin Horstmann, Manfred Jücker

**Affiliations:** 1 Institute of Biochemistry and Signal Transduction, Center for Experimental Medicine, University Medical Center Hamburg-Eppendorf, Martinistr. 52, 20246 Hamburg, Germany; 2 Research Institute Children’s Cancer Center Hamburg, Hamburg and Department of Pediatric Oncology and Hematology, University Medical Center, 20246 Hamburg, Germany; 3 Department of Otorhinolaryngology, University Medical Center Hamburg-Eppendorf, 20246 Hamburg, Germany; 4 UCCH Kinomics Core Facility, University Cancer Center Hamburg (UCCH), University Medical Center Hamburg-Eppendorf, 20246 Hamburg, Germany; 5 Center for Oncology, Clinic for Radiation Therapy and Radiation Oncology, University Medical Center Hamburg-Eppendorf, 20246 Hamburg, Germany; 6 Research Department Cell and Gene Therapy, Department of Stem Cell Transplantation, University Medical Center Hamburg-Eppendorf, 20246 Hamburg, Germany; 7 Institute for Clinical Chemistry, University Hospital Köln, 50937 Cologne, Germany

**Keywords:** SHIP1, T-ALL, PI3K/AKT/mTOR-signaling pathway, NTRK, PDGFR

## Abstract

Acute lymphoblastic leukemia (ALL) is the most common cause of cancer-related death in children. Despite significantly increased chances of cure, especially for high-risk ALL patients, it still represents a poor prognosis for a substantial fraction of patients. Misregulated proteins in central switching points of the cellular signaling pathways represent potentially important therapeutic targets. Recently, the inositol phosphatase SHIP1 (SH2-containing inositol 5-phosphatase) has been considered as a tumor suppressor in leukemia. SHIP1 serves as an important negative regulator of the PI3K/AKT signaling pathway, which is frequently constitutively activated in primary T-ALL. In contrast to other reports, we show for the first time that SHIP1 has not been lost in T-ALL cells, but is strongly downregulated. Reduced expression of SHIP1 leads to an increased activation of the PI3K/AKT signaling pathway. SHIP1-mRNA expression is frequently reduced in primary T-ALL samples, which is recapitulated by the decrease in SHIP1 expression at the protein level in seven out of eight available T-ALL patient samples. In addition, we investigated the change in the activity profile of tyrosine and serine/threonine kinases after the restoration of SHIP1 expression in Jurkat T-ALL cells. The tyrosine kinase receptor subfamilies of NTRK and PDGFR, which are upregulated in T-ALL subgroups with low SHIP1 expression, are significantly disabled after SHIP1 reconstitution. Lentiviral-mediated reconstitution of SHIP1 expression in Jurkat cells points to a decreased cellular proliferation upon transplantation into NSG mice in comparison to the control cohort. Together, our findings will help to elucidate the complex network of cell signaling proteins, further support a functional role for SHIP1 as tumor suppressor in T-ALL and, much more importantly, show that full-length SHIP1 is expressed in T-ALL samples.

## 1. Introduction

Leukemia is the most common childhood cancer. Acute lymphoblastic leukemia (ALL) accounts for about 77% of childhood and adolescent leukemia cases [1]. The T-ALL (15% of the ALL of childhood) subgroup has a high risk of relapse, so in consequence this immunophenotype represents an important risk factor for therapy of ALL [2]. The PI3K/AKT/mTOR signaling pathway is of particular interest for targeted therapy of ALL and plays a key role in cancer therapy because of its frequent overactivation in most tumors [3]. The inositol phosphatase SHIP1 (src homology 2 domain-containing inositol phosphatase 1) possesses important functions as a negative regulator of the PI3K/AKT signaling pathway. The regulatory function of SHIP1 is mediated by its inositol 5-phosphatase activity, catalyzing dephosphorylation of phosphoinositides (PI) and inositol phosphates (IP) at the D5 position of the inositol ring. Regulation of phosphoinositide- and inositol phosphate-mediated cellular processes by SHIP1 (especially the conversion of PtdIns(3,4,5)P_3_ to PI(3,4)P_2_ and Ins(1,3,4,5)P_4_ to Ins(1,3,4)P_3_) has been extensively studied [4,5,6,7]. A constitutive activation of AKT can be demonstrated in 87% (21/24) of patients with a T-ALL [8]. The constitutive activation of the AKT signaling pathway negatively influences the response to therapeutic treatments, correlates with drug resistance and is associated with a poor prognosis for ALL patients [9,10,11]. In addition, the AKT/mTOR signaling pathway plays an important role in the progression and survival of T-ALL clones [12]. With regard to the PI3K/AKT signaling pathway, mutations of AKT (isoform 1) and PTEN can be identified for T-ALL [13,14,15]. Mutations of the *INPP5D* gene (SHIP1) in hematopoietic and lymphoid cells are rare (107 mutations out of 5936 primary tissue samples tested, corresponding to 1.8% of all tested cases) [16]. In patients with acute myeloid leukemia (AML), some mutations in the *INPP5D* gene encoding SHIP1 have been described, thereby implicating mutated SHIP1 in the pathogenesis of AML [17,18,19,20,21]. Mutations of the *INPP5D* gene can be found only in one T-ALL patient (SHIP1-T1185S) out of 528 samples (0.19%), according to the COSMIC database [16].

A large number of experimental results strongly suggest that SHIP1 is a tumor suppressor that is involved in leukemogenesis, due to reduced expression and/or reduced enzymatic activity [22,23,24,25,26,27]. The relevance of this study is confirmed by the observation that the inositol phosphatase PTEN, which is a tumor suppressor, is downregulated in two thirds of primary T-ALL samples examined [28]. According to the literature, the Jurkat T-ALL cell line does not express PTEN or SHIP1 [28]. As a result, Jurkat T-ALL cells show a very high level of PtdIns(3,4,5)P_3_ and associated with this a very strong AKT activation [29].

In this study, we analyze in detail the loss of SHIP1 expression at the mRNA and protein level and identify a variety of proteins whose activity is differentially regulated after restoration of SHIP1 in Jurkat SHIP1-null T-ALL cells. We demonstrate for the first time that SHIP1-expressing Jurkat cells exhibit a significantly decreased proliferation in a xenotransplantation mouse model, suggesting a tumor suppressor function of SHIP1 in T-ALL.

## 2. Material and Methods

### 2.1. Patient Material

Bone marrow and peripheral blood samples were obtained after informed consent of parents or guardians from children diagnosed with T-ALL who had been enrolled in the multi-center CoALL studies -92, -97 and -03 [30,31]. The trials achieved approval by the ethics committee of the City of Hamburg (No. 2077; 12 August 2003) and institutional review boards of participating trial centers, and they were conducted according to the principles of the Declaration of Helsinki. Material (buffy coat) from healthy donors whose personal data are not known to us was made available as part of the surplus material from the transfusion medicine department of the UKE, according to a statement by the Central Ethics Committee of the BÄK (federal medical association): “The (further) use of human body materials for medical research purposes (20 February 2003)”.

CD3-positive T cells were isolated from fresh peripheral blood mononuclear cells of healthy donors using EasySep Human CD3 positive selection kit (STEMCELL Technologies) with the associated EasySep magnet (STEMCELL Technologies) for the selection of magnetic particles. The purification was carried out according to the manufacturer’s instructions.

### 2.2. Construction of Plasmids

LeGO-iG2-Puro^+^ SHIP1 vector was generated as previously described [32]. The vector was modified by using QuikChange site-directed mutagenesis [33]. The LeGO-iG2-Puro+ SHIP1 vector was used as template. The following primer pair was used:

SHIP1 D672A

forward 5′-TTGCCTTCCTGGTGTGCCCGAGTCCTCTGGAAG-3′

reverse 5′-CTTCCAGAGGACTCGGGCACACCAGGAAGGCAA-3′

 

SHIP1 Y864A

forward 5′-ACGAGGGAGAAGCTCGCTGACTTTGTGAAGACG-3′

reverse 5′-CGTCTTCACAAAGTCAGCGAGCTTCTCCCTCGT-3′

### 2.3. Lentiviral Transduction

The generation of pseudotyped lentiviruses and transduction were performed as previously described [34]. The 293-T cells were co-transfected with the plasmids Gag-Pol (HIV-1 GAG/POL), Rev (HIV1gp6), VSV-G env and target vector DNA. Target cells were seeded at a density of 5 × 10^5^ cells per well of a 6-well plate. Two ml of fresh culture medium and 2 mL of virus supernatant were added. Transduced cells were selected by the addition of puromycin (Sigma-Aldrich, Taufkirchen, Germany) to the culture medium for at least 1 week before experiments were carried out. 

### 2.4. Cell Culture

All cells were cultivated in tissue culture bottles at 37 °C and 5% CO_2_ according to the DSMZ. The respective medium was mixed with FCS and 1% penicillin/streptomycin (complete medium). The used cells were: Reh (B-ALL; DSMZ No. ACC22), Jurkat (T-ALL; DSMZ No. ACC282), CCRF-CEM (T-ALL; DSMZ No. ACC240), and NCI-H1299 (carcinoma; ATCC No. CRL-5803).

### 2.5. Jurkat Tet-On Cells

Jurkat is a human T-cell line established from a patient with acute lymphocytic leukemia [35]. Jurkat Tet-On cells carrying the reverse transactivator (BD Clontech, Heidelberg, Germany) were cultured in Roswell Park Memorial Institute (RPMI) 1640 Medium, supplemented with GlutaMAX™ I, 25 mM HEPES (Invitrogen, Karlsruhe, Germany), 10% *v*/*v* fetal calf serum (FCS), 1% penicillin-streptomycin solution and 1 mM sodium pyruvate at 37 °C with 5% CO_2_. Jurkat Tet-On cells were under the control of a tetracycline inducible response element (TRE). Individual clones were selected in hygromycine and cloned by limiting dilution, as described by Horn et al. [11]. 

#### Microarray Analysis

Total RNA of 79 T-ALL samples was prepared using Trizol for isolation, phenol chloroform isoamylalcohol for purification and isopropanol for precipitation. Biotinylated cRNAs were prepared according to the Affymetrix One Cycle Protocol, following fragmentation; 12.5 ng of cRNA were hybridized for 16 h at 45 °C on GeneChip Human Genome U133 Plus 2.0 Array. GeneChips were washed and stained in the Affymetrix Fluidics Station 450 and scanned using the Affymetrix GeneChip Scanner 3000 7G. Data were analyzed with Microarray Suite version 5.0 (MAS 5.0) using Affymetrix default analysis settings and target value scaling as the normalization method. The trimmed mean target intensity of each array was set to a value of 200 (NCBI GEO Series: GSE42038) [36].

### 2.6. Gene Expression and Bioinformatic Analysis

RNA-seq data of 106 T-ALL samples were taken from the data set of Dai et al. [37]. Gene expression analysis was carried out on TPM data. Patient samples were ordered according to their molecular subgroup affiliation. The genes of interest were selected according to their association with the AKT signaling pathway and to their association with the T-ALL subgroups (G1–G10) defined by [37]. GSEA (v3.0) software [38] was used for gene set enrichment analysis using the MSigDB Hallmark [39] gene set. For input, gene lists were obtained by the mean fold change between the molecular subgroup G10 and all other subgroups. 

### 2.7. Western Blot Analysis

Western blot analysis was performed as described previously [40]. Protein lysates were prepared by lysing cells in trichloroacetic acid. Proteins were analyzed by SDS–polyacrylamide gel electrophoresis and transferred to the nitrocellulose membrane. In addition, Ponceau S staining of the membrane was performed (loading control). Subsequently, the membrane was hybridized with either rat anti-HA High-Affinity antibody (Roche; 3F10; detects SHIP1 only after lentiviral transduction), mouse anti-SHIP1 antibody (P1C1; Santa Cruz; detects endogenous SHIP1, including after lentiviral transduction), mouse anti-HSC70 antibody (B-6) (both Santa Cruz), rabbit anti-MAPK antibody (9212; Cell Signaling), rabbit anti-phospho-AKT antibody (9018; Cell Signaling), rabbit anti-pan-AKT (11E7; Cell Signaling), rabbit anti-phospho-GSK3β-S9 (9336; Cell Signaling), rabbit anti-phospho-S6-ribosomal protein S240/244 (2215; Cell Signaling) and mouse anti-GAPDH antibody (32233; Santa Cruz). Further antibodies used were anti-mouse IgG HRP-conjugated, anti-rabbit IgG HRP-conjugated (both Cell Signaling) and goat anti-rat IgG HRP (Santa Cruz). Subsequently, protein expression was quantified using LAS-3000 Imager from Fuji (Raytest). 

### 2.8. Functional Kinome Profiling

Functional kinome profiling was used to analyze the activity of protein tyrosine kinases (PTK) and serine/threonine kinases (STK), as described previously [41,42]. Therefore, whole cell lysates were generated using MPER Mammalian Extraction Buffer (Thermo Fisher Scientific, Darmstadt, Germany) with Halt Phosphatase Inhibitor and EDTA-free Halt Protease Inhibitor Cocktail (Pierce Biotechnology, Rockford, IL, USA). The profiling was performed using the PamStation12 (UCCH Kinomics Core Facility, UKE, Hamburg, Germany) and corresponding PTK and STK microarrays (PamChip), according to the manufacturer’s protocols (PamGene, Hertogenbosch, The Netherlands). Lysate containing 5 µg of protein (PTK) or 1 µg (STK) and 400 µM ATP was applied to each array. Phosphorylation of the kinase-specific peptide sequences was detected using either fluorescein-labelled anti-phosphotyrosine antibodies (PTK) or anti-phospho-Ser/Thr antibodies, followed by a secondary polyclonal swine anti-rabbit Immunoglobulin-FITC antibody (STK; PamGene International, the Netherlands). Analysis of the intensity was conducted with the Evolve software v. 1.0 (PamGene, The Netherlands) and for further analysis, the intensities were log2-transformed and proceeded with using the BioNavigator software v. 6.0 (BN6, PamGene, the Netherlands).

### 2.9. RNA Isolation, cDNA Synthesis and Real-Time Quantitative PCR (qPCR)

RNA isolation, cDNA synthesis and real-time quantitative PCR (qPCR) were performed as described previously [40]. Total RNA was isolated with the Direct-Zol RNA MiniPrep Kit (ZYMO Research) following the manufacturer’s instruction. First-strand cDNA was synthesized with the Promega M-MLV Reverse Transcriptase (Promega), following the manufacturer’s instruction. Sense and antisense oligonucleotide primers for amplification of mRNAs of human SHIP1 and the housekeeping genes GAPDH were designed as follows: 

HA-SHIP1-FP 5′-CCTATGACGTGCCCGACTATGC-3′

HA-SHIP1-RP 5′-AGCGGCACAGGGTATTGCAGATGGGTC-3′

SHIP1-3′-UTR-FP 5′-GGAAATCAGCTCCTATTCTCCA-3′

SHIP1-3′-UTR-RP 5′-CACACACCACTGGATTTAGCTC-3′

GAPDH-FP: 5′-GAGTCAACGGATTTGGTCGT-3′

GAPDH-RP: 5′-TTGATTTTGGAGGGATCTCG-3′

Oligonucleotide primers were obtained from Eurofins MWG (Ebersberg, Germany). LightCycler Real-Time PCR reactions and data analysis were performed on a LightCycler system (version 3.5) and LightCycler detection software, according to the instructions of the manufacturer (Roche). 

### 2.10. Flow Cytometry

Flow cytometry analyses were performed on FACSCanto (BD Biosciences) and CytoFlex (Beckman Coulter). For the analysis of stained cells, 1 × 10^5^ cells were taken up in 400 µL PBS with 2% FCS and 2 mM EDTA and incubated according to the manufacturer’s instructions with the corresponding fluorescence-coupled antibody for 30 min in the dark. Then the cells were washed three times, taken up in 400 μL fresh PBS with 2% FCS and 2 mM EDTA, and transferred into a 5 mL round-bottom tube. 

### 2.11. Determination of Cell Growth

The Neubauer hemocytometer is used for the quantification of the cell number in a suspension. For determination of cell growth, 3 × 10^5^ cells were seeded and allowed to grow for the indicated time in a 6-well dish. The cells were incubated during this time at 37 °C in a humidified atmosphere. Subsequently, the cells were stained with trypan blue and counted manually with a Neubauer chamber on a light microscope and, in addition to that, measured automatically in the Countess II cell counter (Thermo Fisher). The cell number was determined on at least the second day after seeding. 

### 2.12. Live-Cell Imaging

The IncuCyte ZOOM system (Sartorius) was used to determine the growth behavior of the examined cells. The system represents a live-cell imaging and analysis platform in an incubator. Cells were seeded in a 96-well plate (Greiner Bio-One, Frickenhausen, Germany) in 200 µL medium and incubated at 37 °C and 5% CO_2_ for at least 48h. The IncuCyte Zoom system was used to take phase-contrast images. Subsequently, phase-contrast images were analyzed by creating a confluence mask with the associated IncuCyte Zoom software, according to the manufacturer’s instructions, and applied to all images. 

For IC50-value determination, cells were treated with various concentrations of the inhibitor. The IC50 value was then used for further cell growth experiments, unless otherwise stated. To inhibit AKT, the cells were treated with MK2206 inhibitor (pan AKT, Merck). For this, the cells were washed with PBS and mixed with the inhibitor in the specified concentration, with fresh medium. DMSO (solvent) was used as a control.

### 2.13. Animal Experiment

Animal experiments were performed in accordance with ARRIVE guidelines and legal regulations (Hamburg TV 43/13 and 68/17) [43]. Non-irradiated, immunodeficient NOD scid gamma mice (NOD.CG-Prkdcscid IL2rgtm1Wji/SzJ; NSG mice) were injected intratibially with 1 × 10^6^ cells each in PBS. To end an animal experiment, the mice were killed by introducing a CO_2_/O_2_ mixture with increasing CO_2_ concentration. A cervical dislocation was performed. In order to isolate the leukemic cells from the mice, bone marrow from the hind legs, as well as the spleen, was prepared. The femur and tibia were cut open at the ends and the bone marrow was isolated by centrifugation (8000× *g* for 30 s). Splenocytes were separated through a sieve (70 μM, BD Falcon) and taken up in the PBS. 

The in vivo imaging was carried out (in cooperation with the core facility “In vivo Optical Imaging” of the University Cancer Center Hamburg (UCCH)) on an IVIS Spectrum in vivo imaging system (PerkinElmer) [36]. For imaging of the NSG mice, the animals were first anesthetized with isoflurane and 100 µg/mouse of the substrate coelenterazine (SYNCHEM) was injected intraperitoneally. The image was recorded 15 min after addition of the substrate. The images were analyzed using the Living Image software (version 4.3.1). The photons per second per animal were measured.

### 2.14. Statistical Analysis

The statistical significance (* *p* ≤ 0.05; ** *p* ≤ 0.01; *** *p* ≤ 0.001) was determined by the Student’s *t*-test. Each experiment was performed at least in triplicate. All figures show mean values, and error bars represent SD, unless indicated otherwise. The statistical analysis data were analyzed using GraphPad Prism (GraphPad Software Inc., San Diego, CA, USA). Gene expression data were analyzed using one-way ANOVA-tests and Benjamini–Hochberg correction for multiple testing. 

## 3. Results

### 3.1. Expression Analysis of SHIP1 in Leukemia Cells of T-ALL Patients

**Figure 1:**
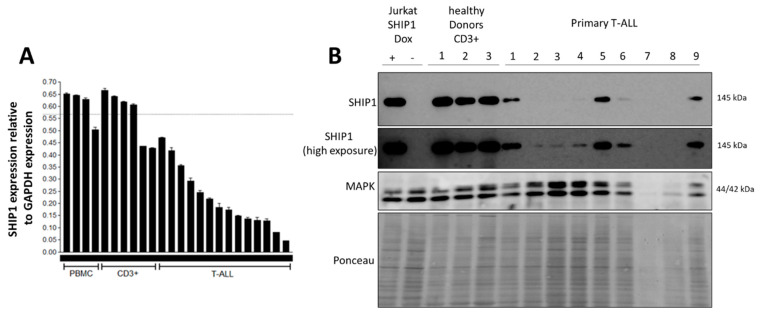
Reduced expression of SHIP1 in primary T-ALL samples. (**A**) SHIP1 mRNA expression was analyzed in peripheral blood mononuclear cells (PBMC) from healthy donors, healthy CD3-enriched (CD3+) cells and primary T-ALL patient samples. (**B**) SHIP1 protein expression was analyzed in Jurkat cells containing a Tet-regulated wild-type SHIP1 cDNA, in healthy CD3-positve cells and in primary T-ALL samples. Ponceau S staining of the membrane was performed as a loading and blotting control.

In order to investigate whether the decrease in SHIP1 expression observed in Jurkat T-ALL cells [28] can also be observed in primary T-ALL cells, we measured SHIP1-mRNA expression using the RNA real-time quantitative PCR (qPCR) technique and SHIP1 protein expression using the Western blot technique. SHIP1-mRNA expression was decreased, but could be detected in all 14 T-ALL patient samples investigated (Figure 1A). In addition, transcriptome studies were carried out using Affymetrix microarray analyzes. An absent call for SHIP1 could be detected in 65 out of 79 examined T-ALL patient samples (82%) (Figure S1 and Table S1). We also examined SHIP1 protein expression in nine primary T-ALL patient samples, in three healthy CD3-positive samples and in the doxycycline SHIP1 inducible Jurkat cell line as control (Figure 1B). One sample showed no adequate protein amount (P7). After high exposure, SHIP1 full-length protein was detected at 145 kDa in seven of eight patient samples (P1, P2, P3, P4, P5, P6 and P9). In summary, and contrast to other reports, we could show for the first time that full-length SHIP1 was expressed in almost all the T-ALL patient samples investigated. However, we have to mention that protein expression was drastically reduced compared to healthy CD3-positive T-cells. We also measured the protein expression of SHIP1 in T-ALL cell lines (Jurkat and CEM) compared to healthy CD3-positive cells (Figure S2). Protein expression of SHIP1 could be identified in the CEM cell line but not in the Jurkat cell line. In addition, the AKT signaling pathway was activated in Jurkat cells (phospho-AKT S473, phospho-S6 S240/244 and phospho-GSK3β S9). We therefore decided to use Jurkat cells for further experiments.

**Figure 2:**
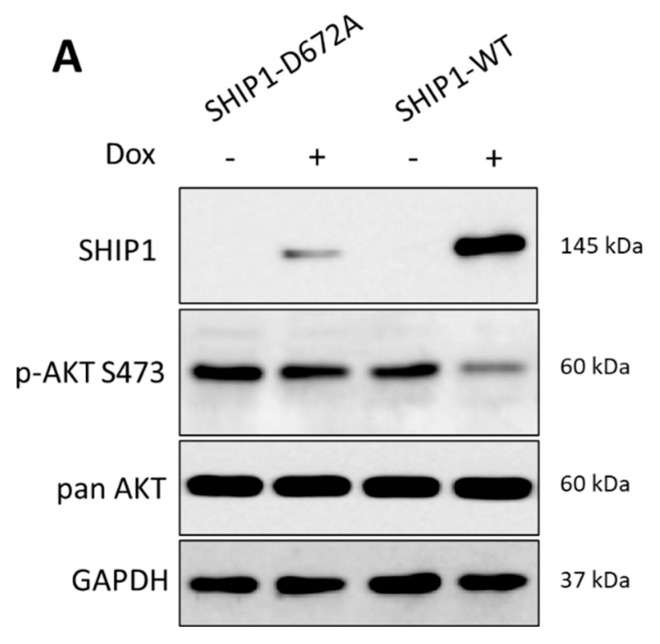

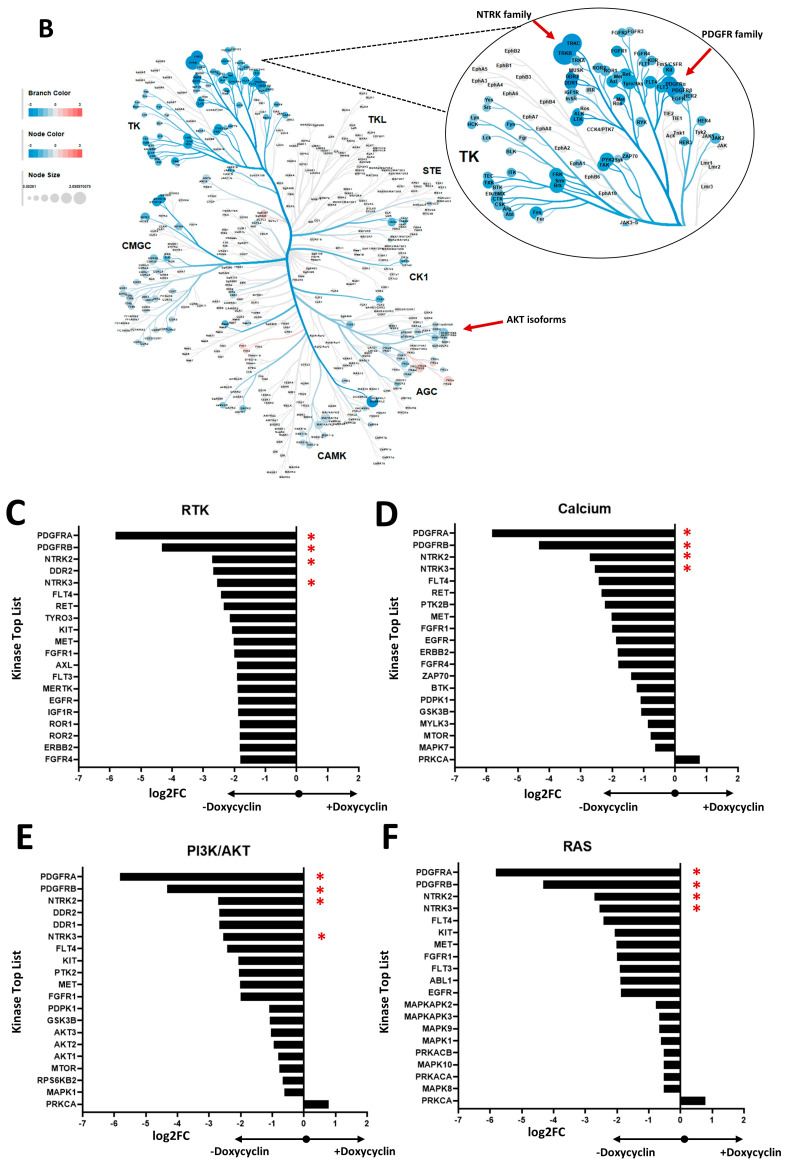

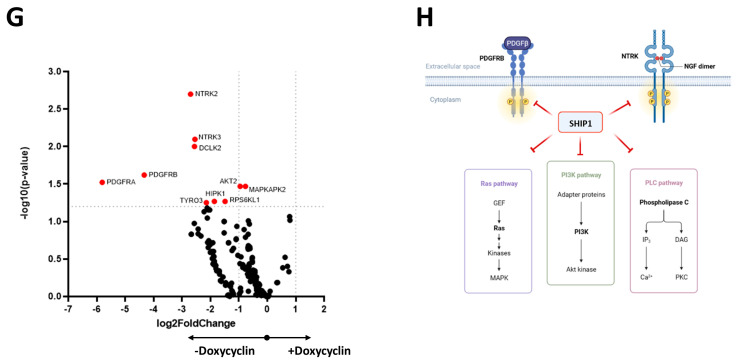
Influence of the reconstitution of SHIP1 on the activity profile of tyrosine and serine/threonine kinases in Jurkat cells. (**A**) Jurkat cells with or without Doxycycline-dependent expression of SHIP1-wt or SHIP1 mutant D672A. Analysis of the influence of SHIP1-wt on the activity profile of tyrosine and serine/threonine kinases in Jurkat cells. (**B**) Kinome tree visualization of the differential kinase activity profile after reconstitution of SHIP1 in Jurkat cells. The influence on the family of tyrosine kinases (TK) is shown enlarged. The NTRK and PDGFR subfamilies are particularly recognizable here. Branch and node color show the influence (kinase statistic) on each kinase after reconstitution of SHIP1. Blue indicates a negative regulation and red indicates a positive regulation after SHIP1 reconstitution. The node size visualizes the influence (specificity score) on the kinase after reconstitution of SHIP1. (**C**–**F**) show the influence of SHIP1 reconstitution on the activity profile of tyrosine and serine/threonine kinases (top list) according to the signal transduction pathway ((**C**): RTK signaling; (**D**): Calcium signaling; (**E**): PI3K/AKT signaling; (**F**): MAPK signaling; red asterisk: same genes). The length of the individual bars corresponds to the strength of the change in phosphorylation. (**G**) Volcano plot visualization of the influence of SHIP1-wt on the activity profile of tyrosine and serine/threonine kinases in Jurkat cells. Significant regulated kinases are shown in red (dots). (**H**) Model of the influence of SHIP1 on the signal transduction process in T-ALL cells. SHIP1 negatively affects phosphorylation and signaling of the PI3K/AKT, RAS/MAPK, and calcium/PLC signaling pathways in T-ALL cells. The receptors of the NTRK and PDGFR subfamilies are of central importance here.

To identify kinases whose activity profile is differentially regulated after restoration of SHIP1 protein expression in Jurkat SHIP1-null T-ALL cells, a doxycycline inducible SHIP1-TetOn expression system (Figure 2A) was used. A kinome tree visualization [44] of the differential kinase activity profile after SHIP1 reconstitution shows that the family of tyrosine kinases (TK) are most affected (Figure 2B). The NTRK and PDGFR subfamilies show the greatest importance for further analysis. In general, the influence of the reconstitution of SHIP1 on the activity profile of serine/threonine- and tyrosine kinases in Jurkat cells showed that SHIP1 reconstitution mainly negatively affects the PI3K/AKT and MAPK signaling pathways, as well as calcium signaling (Figure 2C–F). The differential regulated kinases were consequently grouped according to their classification in known signaling pathways (PI3K/AKT: KEGG hsa04151, MAPK: KEGG hsa04014, and calcium: KEGG hsa04020). PDGFRA/B and NTRK2/3 were not only found in the RTK group, but also in the PI3K/AKT, MAPK and calcium signaling pathway groups (marked with red asterisks), and show the strongest log2 fold change after SHIP1 reconstitution (>2 log2FC).

In addition to this, a volcano plot visualization of the influence of SHIP1 reconstitution on the activity profile of tyrosine and serine/threonine kinases in Jurkat cells demonstrates that PDGFRA/B, NTRK2/3, AKT2, TYRO3, HIPK1, RPS6KL1 and MAPKAPK2 are significantly regulated (red dots; Figure 2G). Based on the activated RTKs PDGFR and NTRK, the signaling pathways of the PI3K/AKT signaling pathway, the MAPK signaling pathway and the PLC/calcium signaling pathway are initiated. In our model, SHIP1 negatively affects phosphorylation and signaling of the PI3K/AKT and RAS/MAPK, and the calcium/PLC signaling pathways in T-ALL cells (Figure 2H). These studies show that the expression of SHIP1 is reduced in a significant number of T-ALL patient samples, and indicate a loss of function of SHIP1 in T-ALL cells, which may have a functional role in the leukemogenesis of T-ALL by influencing different signaling pathways (PI3K/AKT, MAPK and PLC).

### 3.2. Investigation of the Reduced SHIP1 Expression in the T-ALL Cell Line Jurkat

**Figure 3:**
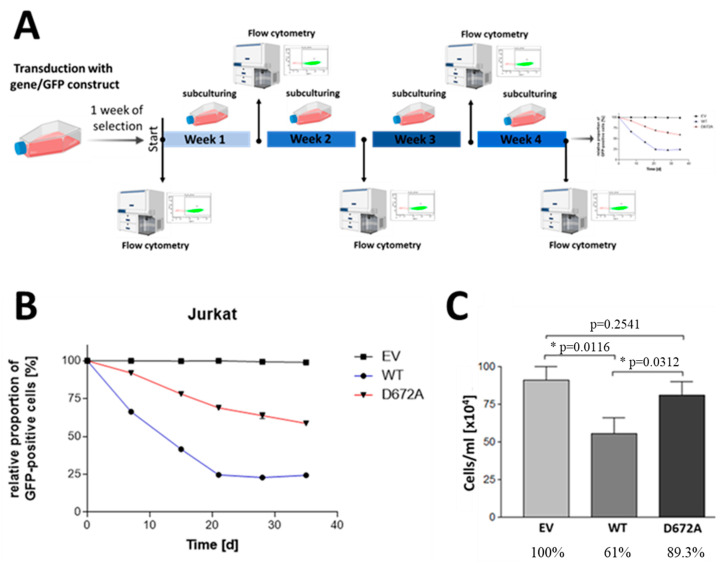
Investigation of the growth behavior of Jurkat cells after reconstitution of SHIP1 expression. (**A**) Schematic of the flow cytometric measurement of cells with SHIP1/GFP constructs. Jurkat cells were transduced with lentiviral vectors encoding SHIP1-wt, SHIP1-D672A and the control vector. The cells were selected with puromycin after transduction for one week. (**B**) Three days after selection, the eGFP-positive cells were examined by flow cytometry, over time. The relative percentage of eGFP-expressing cells is shown. The results were normalized to the first measuring point. (**C**) The stable SHIP1-wt and SHIP1-D672A-expressing Jurkat cells were each seeded with the control vector-expressing Jurkat cells in a cell density of 3 × 10^5^ cells/well in a 6-well dish with 2 mL medium; 48 h after seeding, the cells were counted, using a hemocytometer. The statistical significance (* *p* ≤ 0.05) was determined by the Student’s *t*-test.

**Figure 4:**
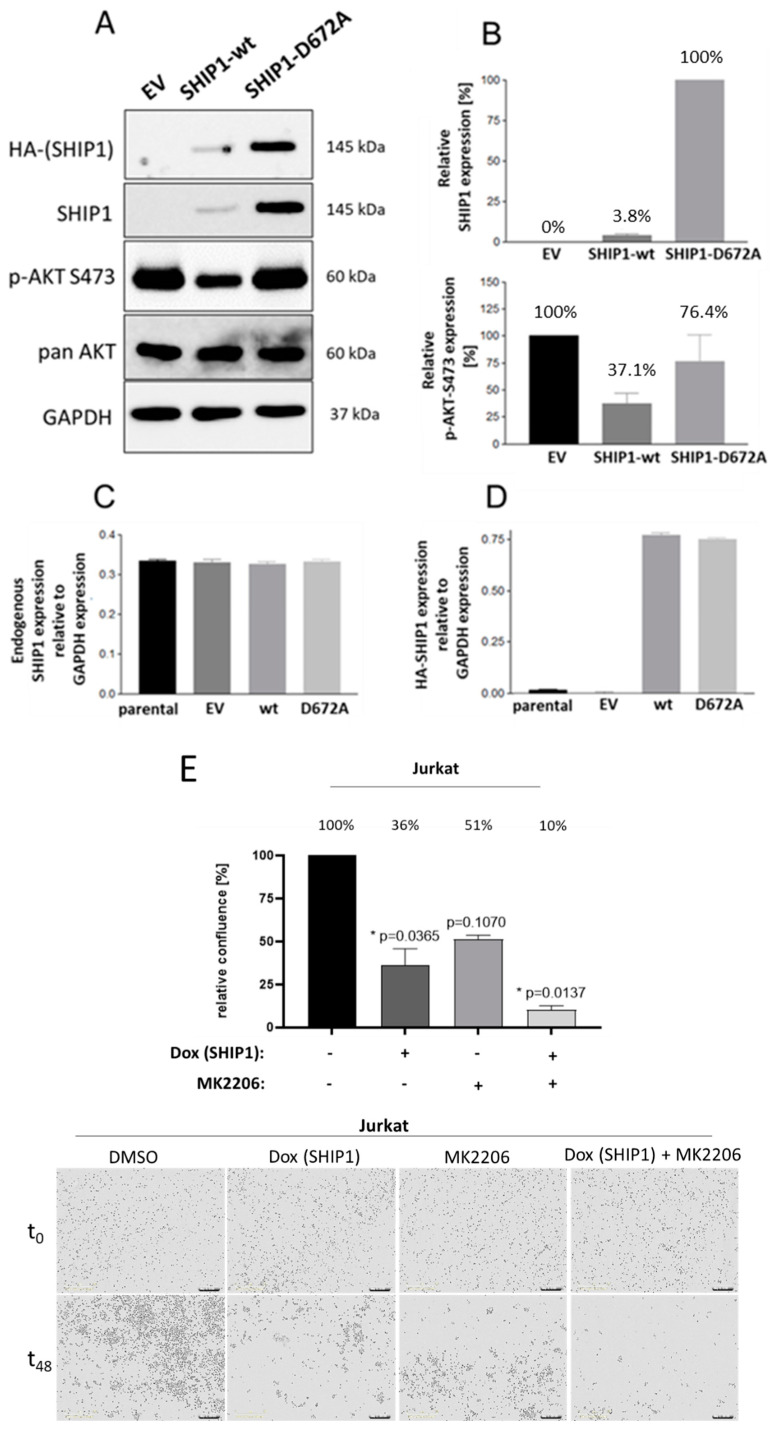
Loss of SHIP1 in Jurkat cells after reconstitution of SHIP1 expression. Jurkat cells were transduced with lentiviral vectors encoding SHIP1-wt, SHIP1-D672A and the control vector. The cells were selected after transduction with puromycin. Two weeks after the transduction, protein lysates were prepared by TCA precipitation of the Jurkat cells with the control vector (EV), the SHIP1-wt (wt) and the SHIP1-D672A mutant (D672A). Lysates were separated by SDS-PAGE, and the proteins were subsequently transferred to a nitrocellulose membrane and detected with specific antibodies (**A**). The relative expression of SHIP1 (wt vs. D672A significant reduction) and p-AKT-S473 (wt vs. EV significant reduction; D672A vs. EV not significant) was determined by normalization to the reference protein GAPDH (**B**). The RNA of the transduced cells was also isolated three weeks after the transduction. The amount of SHIP1 mRNA was determined from the respective cDNA using RT-qPCR. The relative expression of the SHIP1 mRNA amount was determined by normalization to the reference gene GAPDH. Both specific oligonucleotides for the endogenous SHIP1 (**C**) and for the transduced SHIP1 (HA tag) (**D**) were used. The mean ± standard deviation from three measurements of the respective sample is shown in each case. (**E**) The effect of SHIP1 reconstitution before and after treatment with the AKT inhibitor MK2206 was investigated by live-cell imaging (* *p* ≤ 0.05). Representative live-cell imaging phase-contrast images are shown below.

Since SHIP1 is downregulated at the mRNA level and at the protein level in primary T-ALL patient samples as well as in the Jurkat cell line, SHIP1 expression was reconstituted in the Jurkat cell line by the use of lentiviral vectors. The effects of the SHIP1 reconstitution in Jurkat cells was then examined. For this purpose, Jurkat cells were transduced with lentiviral vectors which code for the SHIP1-wt as well as for an enzymatically reduced SHIP1 mutant D672A [45]. The empty vector was also used as a control. The success of the transduction was measured using the enhanced green fluorescent protein (eGFP), which is also introduced into the genome of the cell by the lentiviral vector. The vector for lentiviral transduction also contains a puromycin resistance cassette, which additionally allows the selection of the transduced cells from the non-transduced cells using the antibiotic puromycin. The eGFP signal of the transduced Jurkat cells was measured over 35 days (Figure 3A,B). Two weeks after the transduction, protein lysates were prepared by TCA precipitation from the Jurkat cells with the control vector (EV), the SHIP1-wt (wt) and the SHIP1-D672A mutant, and analyzed using the Western blot technique (Figure 4A,B). In parallel with the processing of the protein lysates, the proliferation of the cells was examined. To determine SHIP1-mediated effects on the proliferation capacity of the transduced cells, cell growth was determined 48 h after the cells were seeded, using a hemocytometer (Figure 3C). Three weeks after transduction, the RNA of the transduced cells was isolated, the cDNA synthesis was carried out, and the SHIP1 expression was measured using RT-qPCR. Specific oligonucleotides were used for the endogenous SHIP1 (Figure 4C) and for the transduced SHIP1 (HA-tag) (Figure 4D).

Figure 3 shows that the relative proportion of eGFP-positive cells decreases substantially with SHIP1-wt (Figure 3B and Figure S3). After three weeks, the relative proportion of eGFP positive cells in these cultures is only 25% of the cells. In contrast, the proportion of eGFP-positive cells in the enzymatically less-active SHIP1-D672A mutant is more than 65% at day 21 and more than 50% of the cells after 35 days. In contrast, the relative proportion of eGFP-positive cells in the control vector shows almost no decrease over time, and remains constant at above 95%. The results of the Western blot analysis (Figure 4) show that after two weeks the relative SHIP1-wt protein level is only 4% of the SHIP1 expression of the D672A mutant. Based on the AKT phosphorylation status of the control vector, the remaining four percent of the SHIP1-wt expression are nevertheless able to significantly reduce the phosphorylation of AKT (S473) to 37%. The enzymatically impaired mutant D672A does not significantly reduce the phosphorylation of AKT (76%). Interestingly, three weeks after the transduction, the SHIP1 mRNA analysis (Figure 4C,D) showed comparable expression levels of both the SHIP1 wild-type and the SHIP1-D672A mutant. As a result, SHIP1-wt expression is downregulated at the protein level, whereas SHIP1 expression remains constant at the mRNA level. In addition, the SHIP1 wild-type expressing cells show a growth disadvantage compared to the vector-expressing control cells (Figure 3). In comparison to the control vector-expressing cells, the SHIP1-wt only grew by 61% two days after seeding. The enzymatically impaired SHIP1 mutant D672A grew by 89% compared to the empty vector control. 

For model (and technical) validation, we measured the same experiments in H1299 SHIP1-null cells (Figures S4–S7). H1299 cells are very suitable as a model cell system, because they have a strong phosphorylation of AKT (S473) and do not express SHIP1 or PTEN protein [46]. H1299 SHIP1-null cells were transduced with the control vector, the SHIP1-wt, the enzymatically reduced SHIP1 mutant D672A and the SHIP1-Y864A mutant. The previously uncharacterized Y864A mutant (the tyrosine residue 864 is located directly C-terminal to the C2 domain of SHIP1) shows a greater reduction in phospho-AKT S473 expression compared to the other cases. In addition, we chose Reh B-ALL cells as a further control cell system (Figures S4–S7) and transduced these cells with the control vector and the SHIP1 wild-type construct.

In contrast to Jurkat cells, H1299 and Reh cells show no obvious reduction in SHIP1 protein expression, and also have a constant population (a relative proportion of GFP-positive cells of over 95% within the three-week observation window) of eGFP-expressing cells after transduction during the indicated time. 

The growth disadvantage of SHIP1-expressing Jurkat cells thus points, in summary to a tumor suppressor function of SHIP1 for T-ALL.

In addition, the effect of SHIP1 reconstitution before and after treatment with the AKT inhibitor MK2206 was investigated by live-cell imaging (Figure 4E and Figure S8). The reconstitution, in combination with the simultaneous inhibition of AKT by 250 nM MK2206, has a clear benefit on the inhibition of cell growth of Jurkat T-ALL cells. Induction of SHIP1 expression alone reduces significantly the cell growth of Jurkat cells. After treatment of Jurkat cells with 250 nM MK2206 alone, cell growth was not significantly reduced.

### 3.3. Xenotransplantation Model of the Consequences of Reconstitution of SHIP1 Expression in T-ALL Cells

In a preliminary experiment, we investigated the growth behavior of Jurkat T-ALL cells in a murine xenograft model. Using Jurkat cells with a luciferase reporter gene integrated for visualization of cell growth in the organism, we determined that Jurkat cells aggressively invade the central nervous system (the brain and along the spinal cord) quite fast (Figure S9A). About three weeks after injection, we terminated the experiment because the mice could no longer walk properly and seemed to have problems, due to an impaired nervous system. After termination of the experiment, we dissected the animal and analyzed the organelles using luciferase in vivo imaging. Both the isolated spleen and the brain showed a strong infiltration of T-ALL cells in the tissue (Figure S9B). We therefore decided to choose a conspicuous behavior of the animal in relation to the central nervous system as an ultimate termination criterion.

**Figure 5:**
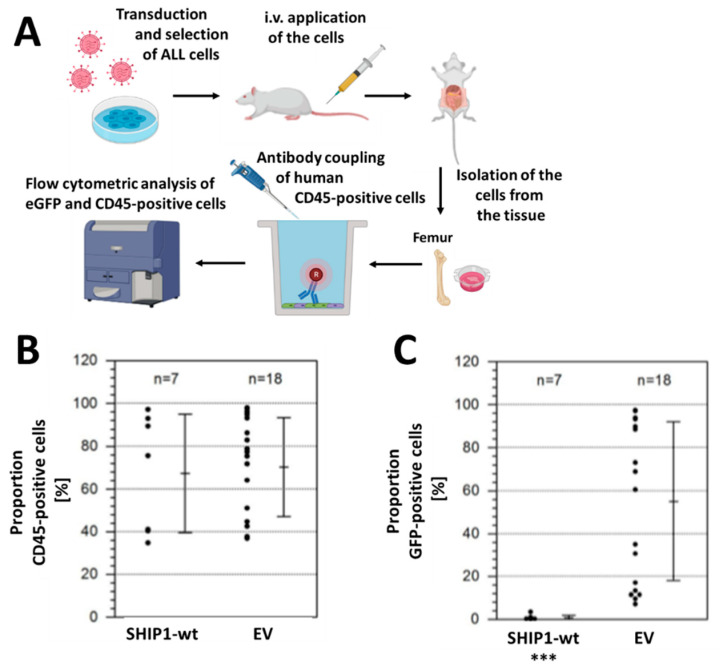
Proportion of hCD45-positive and eGFP-positive cells in the bone marrow of xenotransplanted mice. (**A**) Xenograft mouse model of SHIP1-wt-expressing Jurkat T-cells in immunodeficient mice (NSG). Immunodeficient mice were intratibially transplanted with 1 × 10^6^ cells of fresh vector control or SHIP1-wt-transduced and selected Jurkat cells. Diseased mice were killed, the bone marrow was extracted from the hind bones, and the cells were stained with an APC-coupled anti-hCD45 antibody in order to be able to identify the human Jurkat cells by using flow cytometry. The bone marrow of the diseased mice was analyzed for engraftment with (**B**) human CD45-positive and (**C**) human CD45/eGFP double positive cells using flow cytometric analysis. The level of significance was evaluated by *t*-test (*** *p* < 0.001). (**A**) was created by the use of Biorender.

Immunodeficient mice were then intratibially transplanted with 1 × 10^6^ cells of fresh vector control or SHIP1-wt-transduced and selected Jurkat cells. According to the termination criteria, the experiment was terminated. The bone marrow was extracted from the hind bones and the cells were stained with an APC-coupled anti-human CD45 (anti-hCD45) antibody in order to specifically identify the human Jurkat cells using flow cytometric analysis (Figure 5A and Figure S10). Additionally, the proportion of eGFP/hCD45 double-positive cells was determined. Figure 5B shows the proportion of human cells in the bone marrow of the mice. The mean chimerism of human cells in both cohorts was approximately 70%. The mean proportion of eGFP-positive cells among the human cells is shown in Figure 5C. Indeed, clear differences were visible. While in the mice transplanted with vector-transduced cells the mean relative proportion of eGFP-positive cells was still greater than 50%, mice transplanted with SHIP1-wt-expressing cells harbored less than 5% eGFP-positive cells. The difference in eGFP-positivity between the two groups was highly significant (*p* < 0.001, two-tailed *t*-test). 

### 3.4. Gene Expression Analysis of INPP5D and Molecular Classification of INPP5D in T-ALL Subgroups

**Figure 6:**
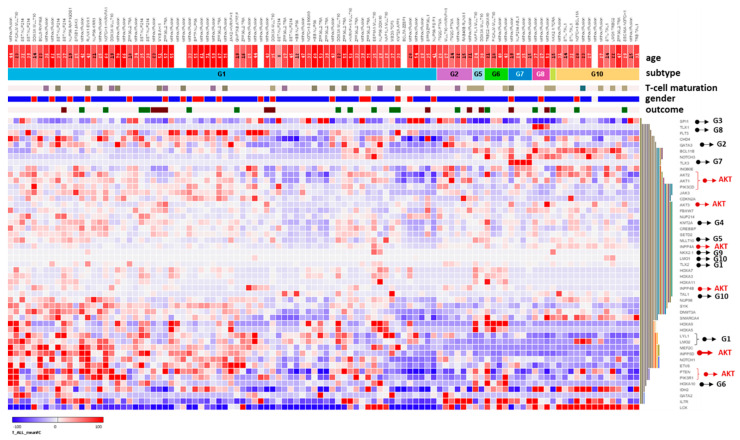

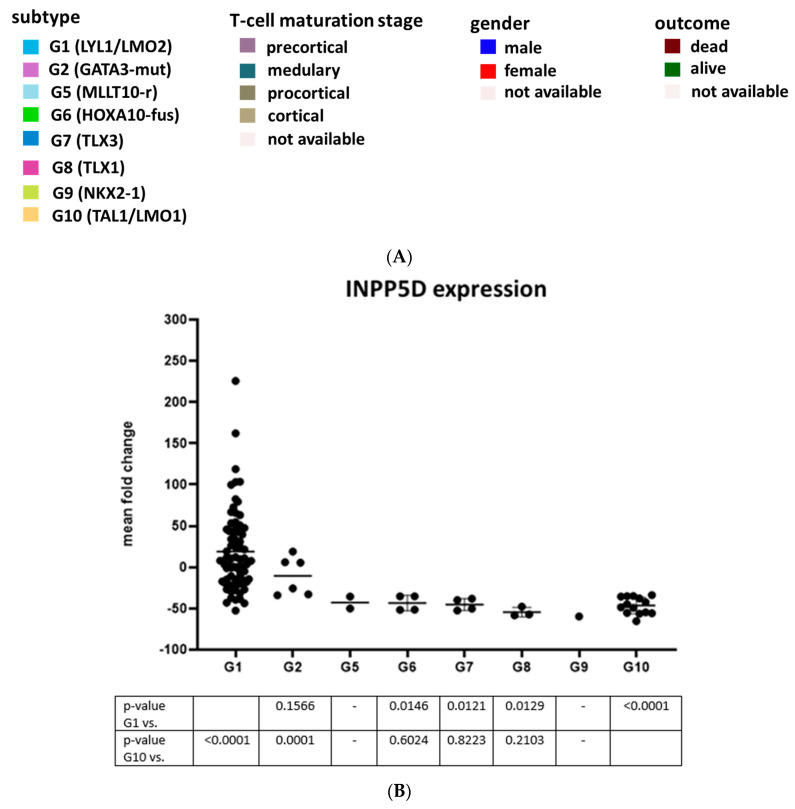

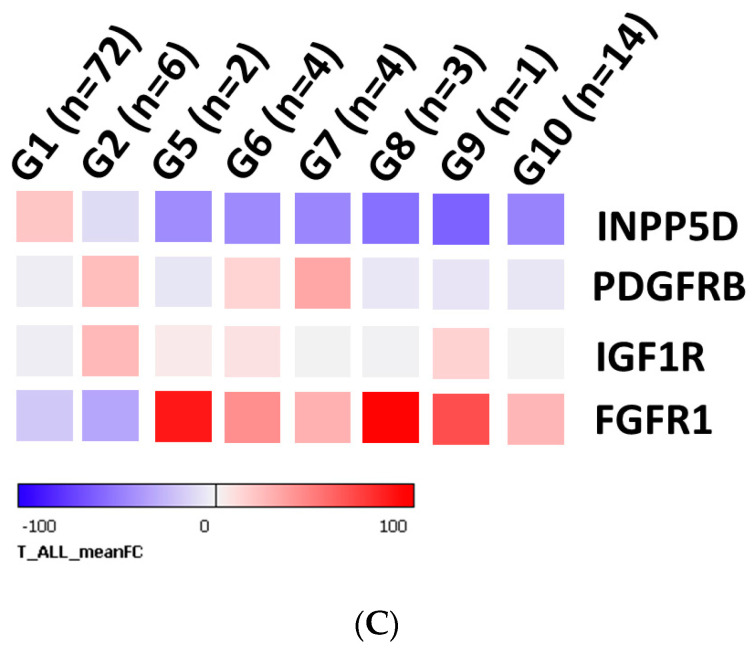
Gene expression analysis of *INPP5D*, the AKT pathway and the molecular subgroups of T-ALL are investigated in transcriptome data of 106 T-ALL patient samples. (**A**) Columns indicate T-ALL patients and clinical information (age, subtype, T-cell maturation stage, gender and clinical outcome), rows represent gene expression (gene expression level (mean fold change) of *INPP5D*, further genes of the AKT pathway and well-known T-ALL defining genes). Patient samples were arranged according to subgroup affiliation. For the gene expression, up- and downregulated genes are shown in the heatmap in red and blue, respectively. Ten subtypes are defined according to the molecular features defined by [37]: G1 (LYL1/LMO2 overexpression), G2 (GATA-3 mutation), G3 (SPI1-fusion), G4 (KMT2A-rearrangement), G5 (MLLT10-rearrangement), G6 (HOXA10-fusion), G7 (TLX3 overexpression), G8 (TLX1 overexpression), G9 (NKX2-1 overexpression) and G10 (TAL1/LMO1 overexpression). *INPP5D* expression is shown separately, below. (**B**) The expression of *INPP5D* in the different subgroups of T-ALL with the associated statistical significance is shown in a dot plot. The statistical significance was determined by the Student’s *t*-test. (**C**) Gene expression pattern (aggregated mean fold change) of the gene expression of INPP5D and selected RTKs by subgroup affiliation (see also Figure S11C). Taken from the data set of [37].

We were interested in whether SHIP1 expression is only downregulated in a specific subgroup of T-ALL. Therefore, we analyzed the gene expression of *INPP5D*, the genes of the AKT pathway (indicated by red arrows) and the marker genes of the molecular subgroups (indicated by black arrows and the corresponding subgroup number) of T-ALL using the transcriptome data of 106 T-ALL patient samples (Figure 6A) [37]. The available patient samples were arranged according to their subgroup affiliation (G1–G10) defined by [37] for G1, G2, G5, G6, G7, G8, G9 and G10. Unfortunately, no samples for G3 and G4 were available. 

Ten subtypes are delimited according to the molecular features: G1 (LYL1/LMO2 overexpression), G2 (GATA-3 mutation), G3 (SPI1-fusion), G4 (KMT2A-rearrangement), G5 (MLLT10-rearrangement), G6 (HOXA10-fusion), G7 (TLX3-overexpression), G8 (TLX1-overexpression), G9 (NKX2-1-overexpression) and G10 (TAL1/LMO1-overexpression) [37]. The genes of interest were ordered according to the Euclidean average hierarchical clustering (on the right). The SHIP1 gene *INPP5D* forms a cluster with the genes of the G1 subgroup (LYL1/LMO2) (Figure 6A). In addition to this, *INPP5D* also forms also a cluster with *NOTCH1* and *ETV6*, and especially with *MEF2C*. We noticed a significant downregulation (blue) of *INPP5D* in the G10 subgroup compared to the G1 (*p* ≤ 0.001) and G2 (*p* = 0.001) subgroups (Figure 6). In contrast, we saw a significant higher expression (red) of *INPP5D* in the G1 subgroup compared to the G6 (*p* = 0.0146), G7 (*p* = 0.0121), G8 (*p* = 0.0129) and G10 (*p* ≤ 0.001) T-ALL subgroups (Figure 6B). 

For an unrestricted exploration of transcription factors involved in SHIP1 regulation in T-ALL, we performed gene set enrichment analysis [38] for the MSigDB Hallmark gene sets [39]. Genes with the highest enrichment in the G10 subgroup were included. Further enrichment of the top transcription factor genes was carried out by positive selection of genes from the human transcription factor gene set [47]. These enriched genes were plotted along with *INPP5D* (Figure S11A). Subsequently, correlation analysis between the different transcription factor genes and *INPP5D* was carried out (Figure S11B). The strongest positive correlation (purple) with *INPP5D* could be observed for *MEF2C* (0.79), *HHEX* (0.76), *ELK3* (0.76), *IRF5* (0.74), *ZNF165* (0.72) and *NFATC2* (0.70). In contrast, the strongest negative correlation (green) with *INPP5D* could be observed for *RORC* (−0.50), *PBX4* (−0.49) and *RAG1* (−0.43). In summary, after clustering and correlation analysis we observed in particular that *INPP5D* forms a cluster and shares a similar expression pattern with *MEF2C* in T-ALL cells (Figure 6A).

Moreover, we examined the gene expression of *INPP5D*, together with the gene expression pattern of the most interesting RTKs of our differential kinase activity profile investigation (Figure 2). Therefore, the genes of interest were selected by variance stabilization and plotted (aggregated mean fold change) according to their subgroup affiliation (Figure 6C and Figure S10C). Interestingly, *PDGFRB* (G2, G6 and G7), *IGF1R* (G2 and G9) and *FGFR1* (G5-G10) showed higher expression in subgroups with low *INPP5D* expression (G1). This strongly suggests a need for inhibition of the corresponding RTKs in the specific subgroups with low *INPP5D* expression, which could compensate for the loss of SHIP1 inhibition of the different signaling cascades in T-ALL cells (PI3K/AKT, MAPK, PLC). 

## 4. Discussion

In the present study, reduced SHIP1 levels were detected on mRNA as well as at the protein level in primary T-ALL cells. Moreover, we detected SHIP1 expression at 145 kDa in seven of eight primary T-ALL patient samples at the protein level. In four cases, SHIP1 expression was only very weakly detectable. Here, we report that weak SHIP1 expression can be identified in T-ALL cells, and this complicates the detection of SHIP1 in T-ALL, as it is often found just below the detection threshold and can easily be missed. The loss of SHIP1 and PTEN as negative regulators of the AKT signaling pathway could presumably play an important role in the development of T-ALL disease.

According to the literature, the Jurkat T-ALL cell line was reported not to express SHIP1 or PTEN protein (e.g., by mutations) [28]. However, other sources report no mutation or report only silent mutations of SHIP1 in Jurkat cells [16]. In connection with the frequent functional loss of PTEN due to mutations [14,48] or via post-transcriptional and post-translational mechanisms [8,49,50], increased AKT activity in T-ALL samples can be observed [51,52,53]. Further studies show that the genetic ablation of PTEN induces rapid cell death in B-ALL cells, while the PTEN deletion in mouse hematopoietic stem cells leads to the development of a T-ALL [54,55]. It was recently shown that SHIP1 leads to a significantly longer survival of the mice in a xenograft transplantation model of acute myeloid leukemia [56]. 

The lost expression of SHIP1 in Jurkat cells could be comparable to the loss of SHIP1 expression in the CML cell line K562. No SHIP1 mutations can be identified for the cell line K562 [28]. Rather, the weak SHIP1 expression can be attributed primarily to the presence of the BCR-ABL fusion protein in K562 cells and primary CML cells [25]. Interestingly, BCR-ABL fusion transcripts can also be detected in the Jurkat T-ALL cell line using a two-stage RT-PCR method [57]. However, it is unclear if expression of BCR-ABL contributes to the loss of SHIP1 in Jurkat cells. In fact, it was recently shown that very low (full-length) SHIP1 expression can be found in Jurkat cells [58]. TCE lysis was an appropriate method for reducing artificial protein degradation by proteases during protein lysis for sensitive detection of SHIP1 expression in T-ALL cells.

The investigation of the transcriptional and translational regulation of SHIP1 after reconstitution of SHIP1 in the Jurkat cell line shows that SHIP1 is degraded quickly at the protein level (Figure 4A). In contrast, SHIP1 expression is stable at the mRNA level. Similar effects can be observed after lentiviral transduction of SHIP1 in the AML cell line UKE1 [56,59]. In contrast, little to no reduction in SHIP1 protein expression was found in two other cell lines (H1299 and Reh), which were handled according to the same routine (Figure S4) [40,59]. In the future, these experiments (determination of the SHIP1 status and reconstitution of SHIP1 expression) should be carried out with other suitable T-ALL cell lines and primary cells. A particular challenge remains the transduction of primary patient material and, in particular, T-ALL cells [60]. Targeted manipulation of primary T-ALL cells would significantly advance research.

Importantly, even weak expression of wild-type SHIP1 protein in Jurkat cells was still sufficient to significantly reduce the phosphorylation status of AKT at S473 by about 60%, when compared to vector-expressing control cells (Figure 4A,B). In agreement with the reduction in phospho-AKT-S473, cellular growth of the wild-type SHIP1-expressing cells was also substantially reduced in comparison to vector controls and cells transduced with an enzymatically less active SHIP1 mutant (D672A) (Figure 3C). The D672A mutant is based on the D675G murine mutant in which a critical aspartic acid in its catalytic domain was replaced with a glycine, which significantly impairs its enzymatic activity (90% inactive) [45].

Jurkat T-ALL cells show an aggressive proliferation behavior in the animal model and also affect the central nervous system. Because of the growth disadvantage of SHIP1-expressing Jurkat cells in vitro, we suggest that the SHIP1-expressing cells were subsequently overgrown by SHIP1-negative cells (Figure 3B), still present in the cultures at minor levels despite previous selection of transduced cells by antibiotic treatment. This could also be the reason why the proportion of green fluorescent cells decreases quickly when measuring the proportion of eGFP positive cells in these cultures using flow cytometry (Figure 3B). In addition, the difference in eGFP-positivity (eGFP/hCD45 double-positive cells) between the SHIP1-expressiong cohort and the control cohort in our xenotransplantation model was highly significant (*p* < 0.001, two-tailed *t*-test) (Figure 5). Overall, the results indicate that the SHIP1-expressing Jurkat T-ALL cells in our animal model show, similar to the in vitro experiments, less growth than cells that do not express SHIP1 protein. These results are supported by previous experiments, showing that SHIP1 plays an inhibitory role in the proliferation of Jurkat cells by the negative regulation of cell cycle transition, at least in part through reduced phosphorylation of the retinoblastoma protein Rb and an increased stability of the cell cycle inhibitor p27Kip1 [11]. Nevertheless, further efforts should be made in the future to investigate the influence of the restoration of SHIP1 on T-ALL cells, in more detail.

By analyzing transcriptome data, we noticed a significant downregulation of *INPP5D* in the G10 T-ALL subgroup compared to the G1 (*p* ≤ 0.001) and G2 (*p* = 0.001) T-ALL subgroups (Figure 6). In contrast, we saw a significantly higher expression of *INPP5D* in the G1 subgroup compared to the G6 (*p* = 0.0146), G7 (*p* = 0.0121), G8 (*p* = 0.0129) and G10 (*p* ≤ 0.001) T-ALL subgroups (Figure 6B). The G10 subgroup is featured in pediatric patients with TAL1/LMO1 overexpression and STIL-TAL1, TAL2 fusions, LMO2 fusions and LMO1 fusions. The G10 subgroup is also characterized by mutations of NOTCH1, FBXW7 and the PI3K-AKT-mTOR signaling pathway (especially PTEN mutations) [37]. In contrast, in the G1 subgroup over 50% of patients were adults. This subgroup is associated with SET-NUP214 and NUP98 fusions and increased expression of LYL1, LMO2, SPI1, MEF2C and HOXA family genes, and shows fewer PI3K-AKT-mTOR mutations. Mutated genes in G1 are transcription factors (e.g., ETV6) and RAS pathway genes (e.g., NRAS, KRAS) [37]. We observed that the SHIP1 gene *INPP5D* formed a cluster with the genes of the G1 subgroup (LYL1/LMO2). In addition to that, *INPP5D* also forms a cluster with *NOTCH1*, *ETV6* and especially with *MEF2C*. Correlation analysis between *INPP5D* and the most enriched transcription factor genes shows the strongest positive correlation for *MEF2C, IRF5, HHEX, ZNF165, ELK3* and *NFATC2* (Figure S11B). ELK3 belongs to the ETS family and ETS transcription factors are activated by MAPK signaling [61]. ETS transcription factors are shown to activate MEF2C [62]. In addition, it was demonstrated that PLC/calcium signaling leads to activation of NFAT and MEF2C with the involvement of ETS transcription factors [63]. Elevated MEF2C expression blocks NOTCH-induced T-cell differentiation and leads to cell survival by upregulation of BCL2 [64]. In contrast, the strongest negative correlation with *INPP5D* could be observed for *RORC* (strong upregulation in G8 and G10), *RAG1* (strong upregulation in G8 and G10) and *PBX4*. Interestingly, it can be shown that RAG1 is downregulated in MEF2C-KO mice [65] and NOTCH1 leads to transcriptional activation of RAG1/2 [66]. Consequently, due to the low SHIP1 expression in different subgroups, the unrestrained signaling of RTKs (e.g., PDGFR, NTRK, IGF1R or FGFR1) could lead to an increased PI3K/AKT signaling (as well as MAPK and calcium signaling), which increases the expression of MEF2C and NFAT and as a result, the transcription of their target genes. On the other hand, it cannot be ruled out that a high MEF2C expression in the G1 subgroup could favor *INPP5D* expression. Therefore, whether *INPP5D* is a direct target of MEF2C or whether there is even a negative feedback of the signaling pathway, should be investigated in more detail in the future.

The frequency of T-ALL patient samples with weakly expressed SHIP1 protein is very high. Thus, there could presumably be a general cause for this effect. A number of deregulated genes with activating mutations or translocations for T-ALL are described in the literature: CDKN2A (61%), CDKN2B (58%), NOTCH1 (50%), TAL1 (30%), and FBXW7 (14%) [14]. It is also known that the NOTCH1 receptor is involved in the downregulation of PTEN [15]. An association of NOTCH1 mutations with FLI-1 overexpression is also described [67]. The transcription factor FLI-1 suppresses the transcription of SHIP1 in erythroleukemia [68]. The constitutive activation of the T-ALL oncogenes NOTCH1 and TAL1 by mutation or translocation leads in many cases to a strong misregulation of the expression of a variety of microRNAs [69,70,71]. Many of these oncogenic miRNAs result in a downregulation of important tumor suppressor genes. Indeed, it has been shown that the miRNAs miR-19b, miR20a, miR-26a, miR-92 and miR-223 together downregulate the expression of PTEN and FBXW7 [71]. NOTCH1 increases the transcription of miR-155 and thus represses the expression of the SHIP1 mRNA [72,73,74]. Jurkat cells also harbor an ITD insertion mutation in the juxtamembrane region of the NOTCH1 receptor, which leads to a high expression level of the ICN1 protein, activation of the Src kinase family and the AKT signaling pathway [75,76,77]. The gene expression data in this work also show a clear, negative correlation between the expression of *INPP5D* (SHIP1) and LCK (especially in G1 vs. G10), the most prominent representative of the Src kinase family in T-cells (Figure 6A). Interestingly, it can be shown that Jurkat cells show high Src kinase expression and PP2 (a Src kinase inhibitor) treatment of the Jurkat cells leads to an increase in the expression of SHIP1 [58]. In summary, NOTCH1, TAL1 and LCK define the G10 subgroup of T-ALL, in which *INPP5D* (SHIP1) shows a strikingly significantly lower expression. In the future, potential clinical treatment by activating SHIP1 and inhibiting AKT could further improve current treatment strategies. The SHIP1 activator AQX-1125 could increase the catalytic activity of SHIP1-expressing cells [78].

In addition, we identified kinases whose activity profile is differentially regulated after restoration of SHIP1 in Jurkat SHIP1-null T-ALL cells. SHIP1 reconstitution mainly negatively affects the PI3K/AKT and MAPK signaling pathways, as well as calcium signaling. PDGFR and NTRK could be identified as central players in this oncogenic signaling cascade, and are negatively regulated after restoration of SHIP1 expression (Figure 2). Interestingly, *PDGFRB* (G2, G6 and G7), *IGF1R* (G2 and G9) and *FGFR1* (G5–G10) show higher expression in subgroups with low *INPP5D* expression (Figure 6B). Our results are confirmed by the observations of the group of De Coninck [79]. They observe high PDGFR-β expression in HOXA and TLX3 T-ALL subgroups compared to normal developing T-cells. Moreover, IGF-1 stimulation leads to activation of the PI3K/AKT signaling pathway, which is important for the regulation of MEF2 function in neurons [80]. This strongly suggests a need for inhibition of the corresponding RTK in the specific subgroups with low *INPP5D* expression, which could compensate for the loss of SHIP1 inhibition of the different signaling cascades (PI3K/AKT, MAPK, PLC) in T-ALL cells. We suggest Ilorasertib, a potent ATP-competitive Aurora-, VEGF- and PDGF- inhibitor. A combinatorial inhibition of mTOR and PDGFRB could represent a promising rationale for the treatment of T-ALL patients with low *INPP5D* expression in the future, since it has been shown that a combinatorial inhibition of the RTK FLT3 and mTOR showed synergistic effects in B-ALL [81].

Moreover, the influence of SHIP1 on the corresponding receptors and their activity at the protein level should be examined and confirmed, in more detail, in the future. In addition to the influence of SHIP1 on the AKT signaling pathway (the focus of this work), the influence of SHIP1 on the RAS signaling pathway (via Shc/Grb2/Dok-1) should also be examined in more detail [82]. Loss of SHIP1 expression could lead to activation of both signaling pathways in T-ALL. Here, experiments targeting phosphatase activity (D672A, G585K and R673Q) and/or the interaction domains and motifs (SH2 domain, PXXP motifs, phosphorylation sites) of SHIP1 [21] could help, and lead to a significant improvement in our understanding of T-ALL signaling. 

## 5. Conclusions

In summary, our results demonstrate that SHIP1-expressing Jurkat T-ALL cells possess significantly reduced growth behavior in our model, and this points strongly to a tumor suppressor function of SHIP1 in acute lymphoblastic cells. Moreover, SHIP1 is expressed in T-ALL cells, but at least in part its expression at the protein level is drastically reduced in Jurkat and other T-ALL cells. Consequently, reconstitution of SHIP1 expression in Jurkat cells is stable at the mRNA level, but not at the protein level. In consequence, we observed a strong reduction in SHIP1 expression in six out of eight primary T-ALL samples. These results further support a functional role of SHIP1 as a tumor suppressor in T-ALL. 

## Data Availability

The datasets used and/or analyzed during the current study are available from the corresponding author on reasonable request.

## Supplementary Materials

The following supporting information can be downloaded at https://www.mdpi.com/article/10.3390/cells12131798/s1, Figure S1: Microarray analysis of SHIP1 mRNA level in T-ALL patient samples; Figure S2: Protein expression of SHIP1 in T-ALL cell lines; Figure S3: Representative flow cytometry gating strategy for analysis of the time course of eGFP positive Jurkat cell populations; Figure S4: Model validation for the expression of SHIP1 in H1299 and Reh cells; Figure S5: Representative flow cytometry gating strategy for analysis of eGFP positive H1299 cell populations after two weeks of puromycin selection; Figure S6: Representative flow cytometry gating strategy for analysis of eGFP positive Reh cell populations after two weeks of puromycin selection; Figure S7: Representative flow cytometry gating strategy for analysis of eGFP positive Jurkat cell populations after puromycin selection; Figure S8: IC50 determination of MK2206 in Jurkat T-ALL cells; Figure S9: Jurkat cells aggressively invade the central nervous system after application in a murine xenograft model for preliminary investigation of the behaviour of T-ALL disease; Figure S10: Representative flow cytometry gating strategy for analysis of eGFP positive human Jurkat cell populations after isolation of the bone marrow of xeno-transplantated mice; Figure S11: Gene expression analysis of transcription factors and RTKs according to molecular subtype in transcriptome data of 106 T-ALL patient samples; Table S1: Overview of SHIP1 expression of microarray data compared to Western Blot data of available T-ALL patient samples.

## Abbreviations

PtdIns(3,4,5)P_3_, phosphatidylinositol-(3,4,5)-trisphosphate; PtdIns(3,4)P_2_, phosphatidylinositol-(3, 4)-bisphosphate; SHIP1, src homology 2 domain-containing inositol phosphatase 1.
