# Supplementary material for: SHIP1 Is Present but Strongly Downregulated in T-ALL, and after Restoration Suppresses Leukemia Growth in a T-ALL Xenotransplantation Mouse Model
Source: Cells. 2023 Jul 6;12(13):1798. doi: 10.3390/cells12131798 (PMC10341211; doi:10.3390/cells12131798)
Supplement: Supplementary file 1 [file cells-12-01798-s001.zip › cells-2444175-supplementary.pdf]

SHIP1 is present, but strongly downregulated in T-ALL and after restoration suppresses leukemia growth in a T-ALL xenotransplantation mouse model

Patrick A. H. Ehm [a, b], Ruth Rietow [a,b], Wiebke Wegner [a], Lara Bußmann [c, d], Malte Kriegs [d, e], Kevin Dierck [b], Stefan Horn [f], Thomas Streichert [g], Martin Horstmann [b], Manfred Jücker [a]

Supplementary data

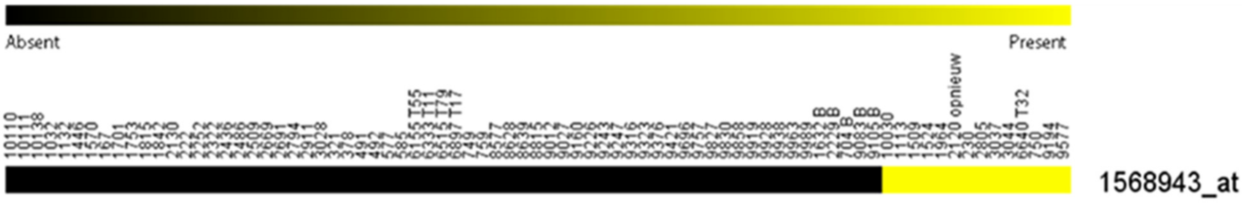

Figure S1: Microarray analysis of SHIP1 mRNA level in T-ALL patient samples. (A) Microarray transcription analysis shows a loss of SHIP1 mRNA expression based on an absent call in 65 of 79 (82%) primary T-ALL samples. Analysis was performed with Affymetrix MAS 5.0 (v.2; Affymetrix) and DMT software using default parameters.

Table S1: Overview of SHIP1 expression of microarray data compared to Western Blot data of available T-ALL patient samples.

Western Blot ID	Western Blot result	Microarray ID	Microarray result
1	present	na	na
2	present	na	na
3	present	na	na
4	present	1032	absent
5	present	2120 opnieuw	present
6	present	na	na
7	absent	2229 B	absent
8	absent	na	na
9	present	2805	present

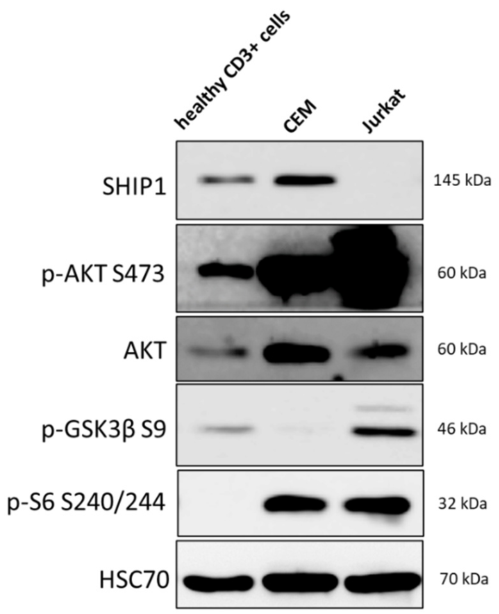

Figure S2. Protein expression of SHIP1 in T-ALL cell lines. Protein expression of SHIP1 and the AKT signaling pathway were analysed in Jurkat and CEM T-ALL cells and in CD3-positve cells of a healthy donor.

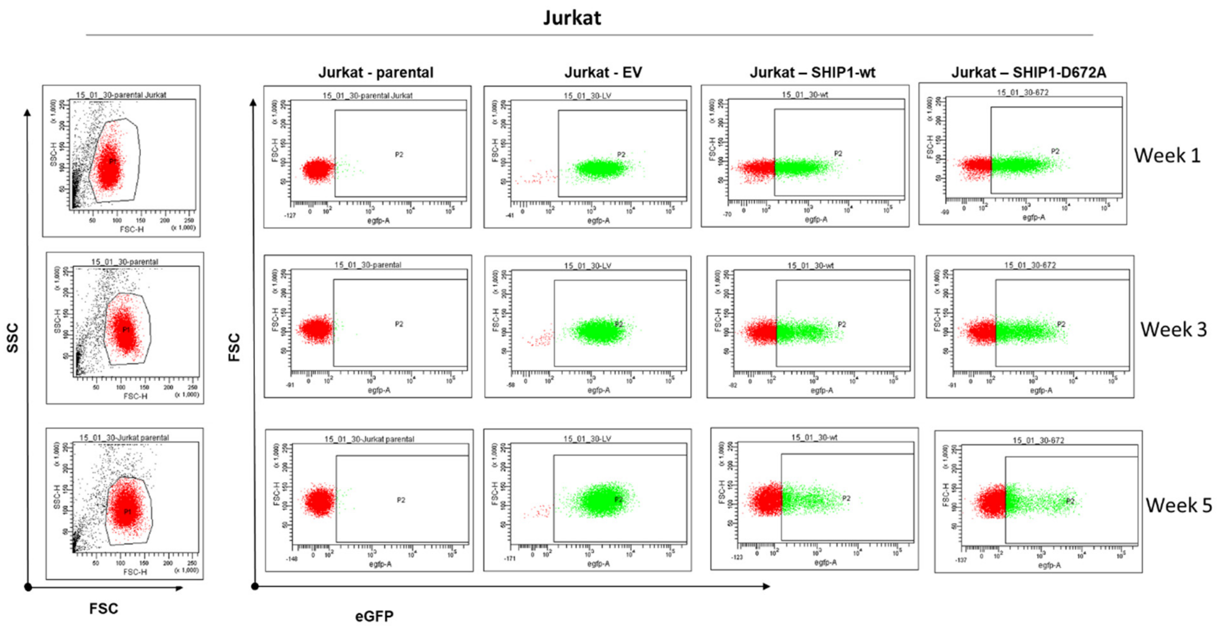

Figure S3: Representative flow cytometry gating strategy for analysis of the time course of eGFP positive Jurkat cell populations. The intact lymphocyte gate was analyzed depending on distinguished FSC vs. SSC properties. Vector transduced cells were identified by eGFP expression at week 1, 3 and 5 after puromycin selection. To indicate the boundaries between eGFP negative and positive populations, gates were set according to parental H1299 populations (without transduction).

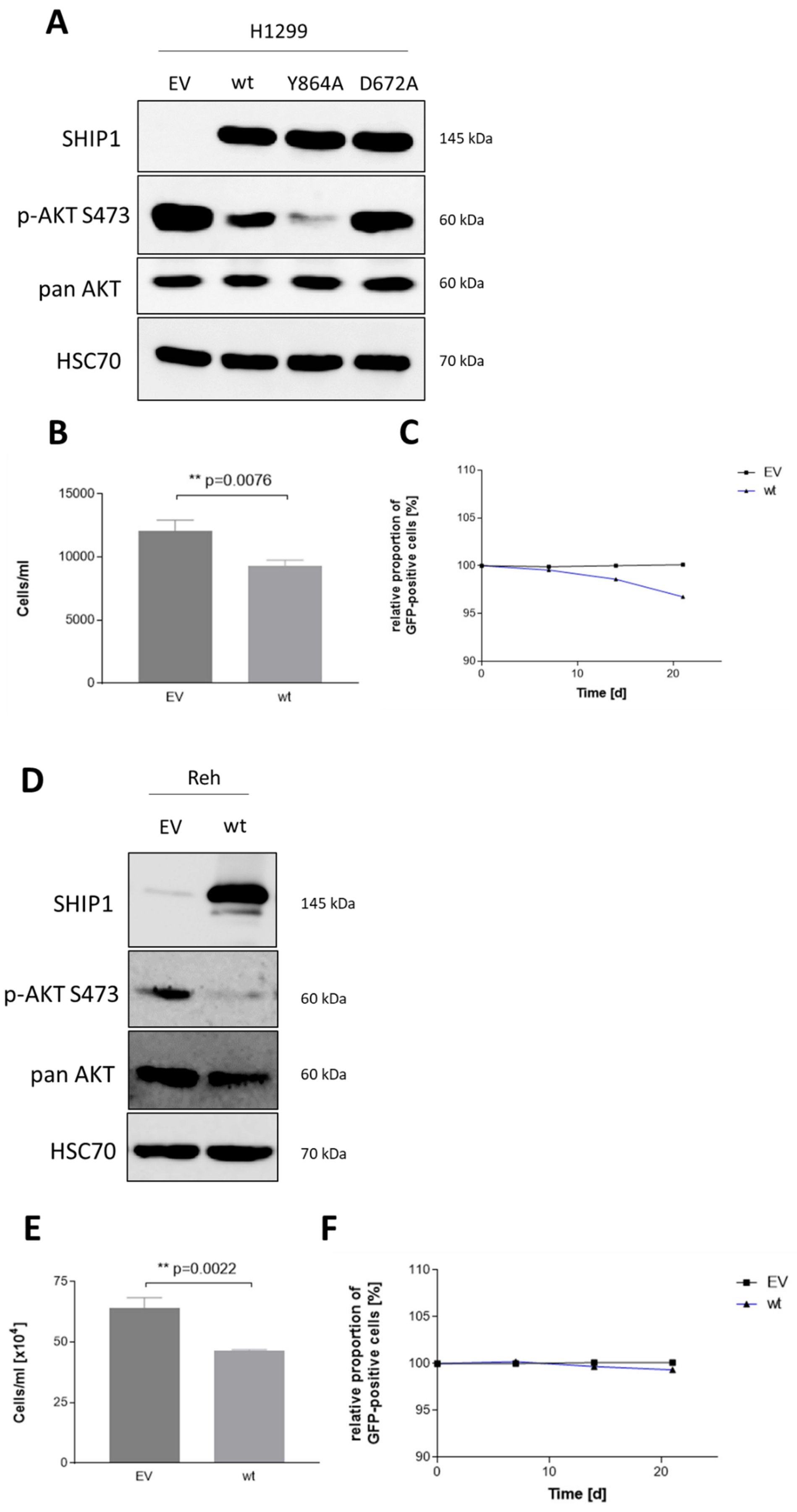
A
H1299
EV
wt
Y864A
D672A
SHIP1
p-AKT S473
pan AKT
HSC70
145 kDa
60 kDa
60 kDa
70 kDa
B
** p=0.0076
Cells/ml
15000
10000
5000
0
EV
wt
C
relative proportion of GFP-positive cells [%]
110
105
100
95
90
0
10
20
Time [d]
EV
wt
D
Reh
EV
wt
SHIP1
p-AKT S473
pan AKT
HSC70
145 kDa
60 kDa
60 kDa
70 kDa
E
** p=0.0022
Cells/ml [x10⁴]
75
50
25
0
EV
wt
F
relative proportion of GFP-positive cells [%]
110
105
100
95
90
0
10
20
Time [d]
EV
wt

Figure S4. Model validation for the expression of SHIP1 in H1299 and Reh cells. (A) H1299 cells were transduced with lentiviral vectors encoding SHIP1-wt, SHIP1-Y864A, SHIP1-D672A and the control vector. The cells were selected after transduction with puromycin. Three weeks after the transduction, protein lysates were prepared by TCA precipitation. Lysates were separated by SDS-PAGE, the proteins were subsequently transferred to a nitrocellulose membrane and detected with specific antibodies. (B) The stable SHIP1 expressing H1299 cells were each seeded with the control vector-expressing H1299 cells in a cell density of 3 x 10^4 cells / well in a 6-well dish with 2 ml Medium. 48 hours after seeding, the cells were counted using a hemocytometer. (C) Three weeks after the transduction, the eGFP-positive SHIP1-wt and control vector cells were examined by flow cytometry over time. The relative percentage of eGFP-expressing cells is shown. The results were normalized to the first measuring point. (D) Reh cells were transduced with lentiviral vectors encoding SHIP1-wt or the control vector. The cells were selected after transduction with puromycin. Three weeks after the transduction, protein lysates were prepared by TCA precipitation. Lysates were separated by SDS-PAGE, the proteins were subsequently transferred to a nitrocellulose membrane and detected with specific antibodies. (E) The stable SHIP1 expressing Reh cells were each seeded with the control vector-expressing Reh cells in a cell density of 3 x 10^5 cells / well in a 6-well dish with 2 ml Medium. 48 hours after seeding, the cells were counted using a hemocytometer. (F) Three weeks after the transduction, the eGFP-positive SHIP1-wt and control vector cells cells were examined by flow cytometry over time. The relative percentage of eGFP-expressing cells is shown. The results were normalized to the first measuring point.

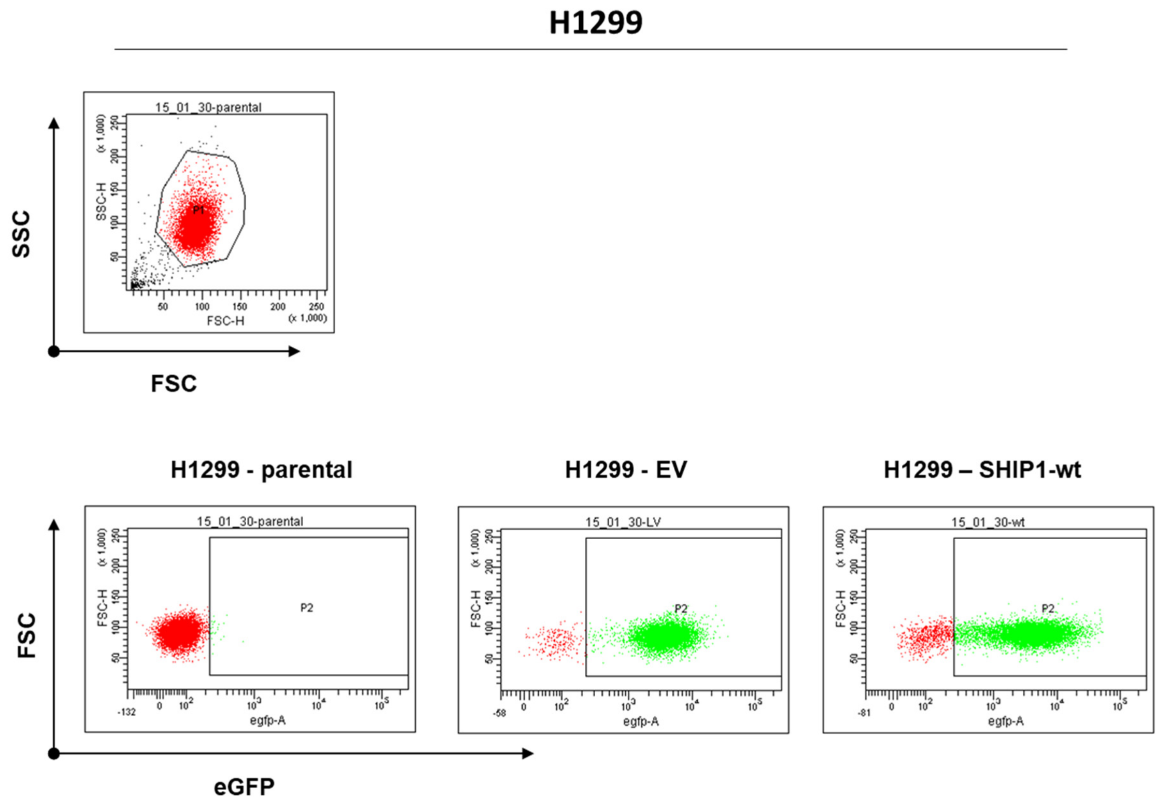

Figure S5: Representative flow cytometry gating strategy for analysis of eGFP positive H1299 cell populations after two weeks of puromycin selection. The intact lymphocyte gate was analyzed depending on distinguished FSC vs. SSC properties. Vector transduced cells were identified by eGFP expression. To indicate the boundaries between eGFP negative and positive populations, gates were set according to parental H1299 populations (without transduction).

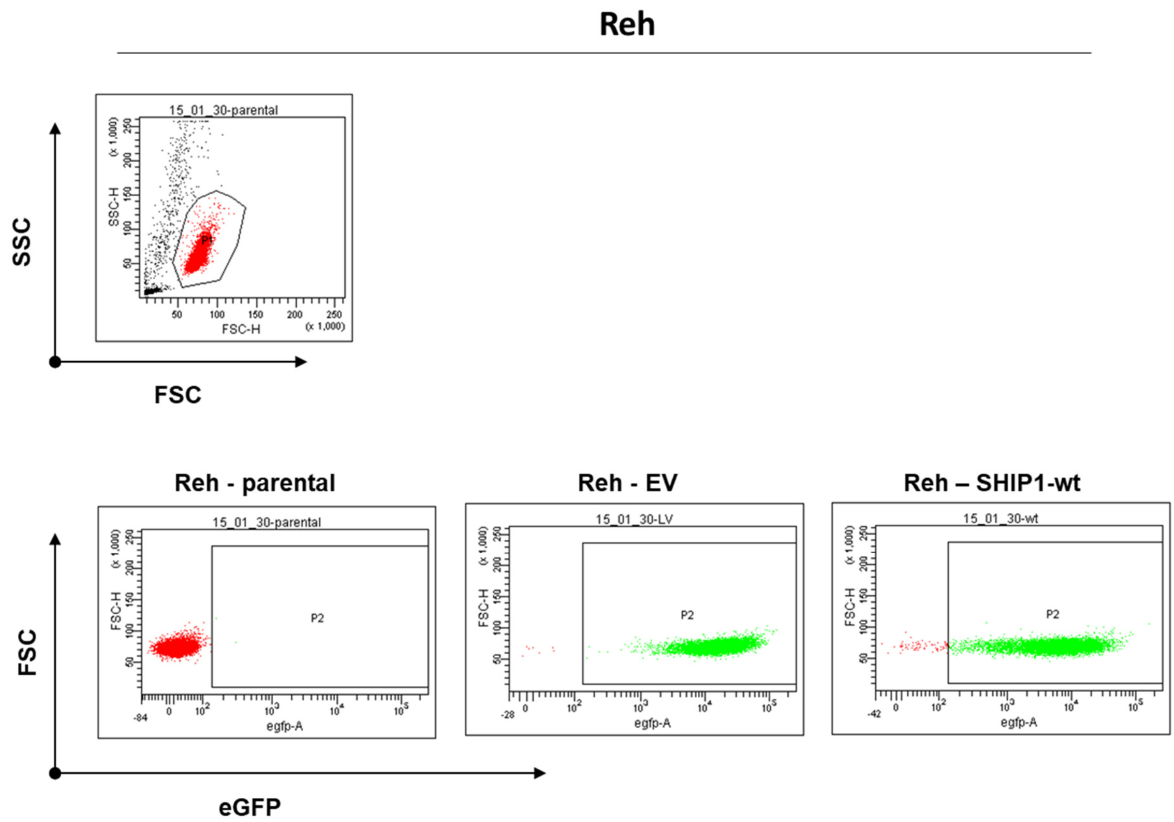

Figure S6: Representative flow cytometry gating strategy for analysis of eGFP positive Reh cell populations after two weeks of puromycin selection. The intact lymphocyte gate was analyzed depending on distinguished FSC vs. SSC properties. Vector transduced cells were identified by eGFP expression. To indicate the boundaries between eGFP negative and positive populations, gates were set according to parental Reh populations (without transduction).

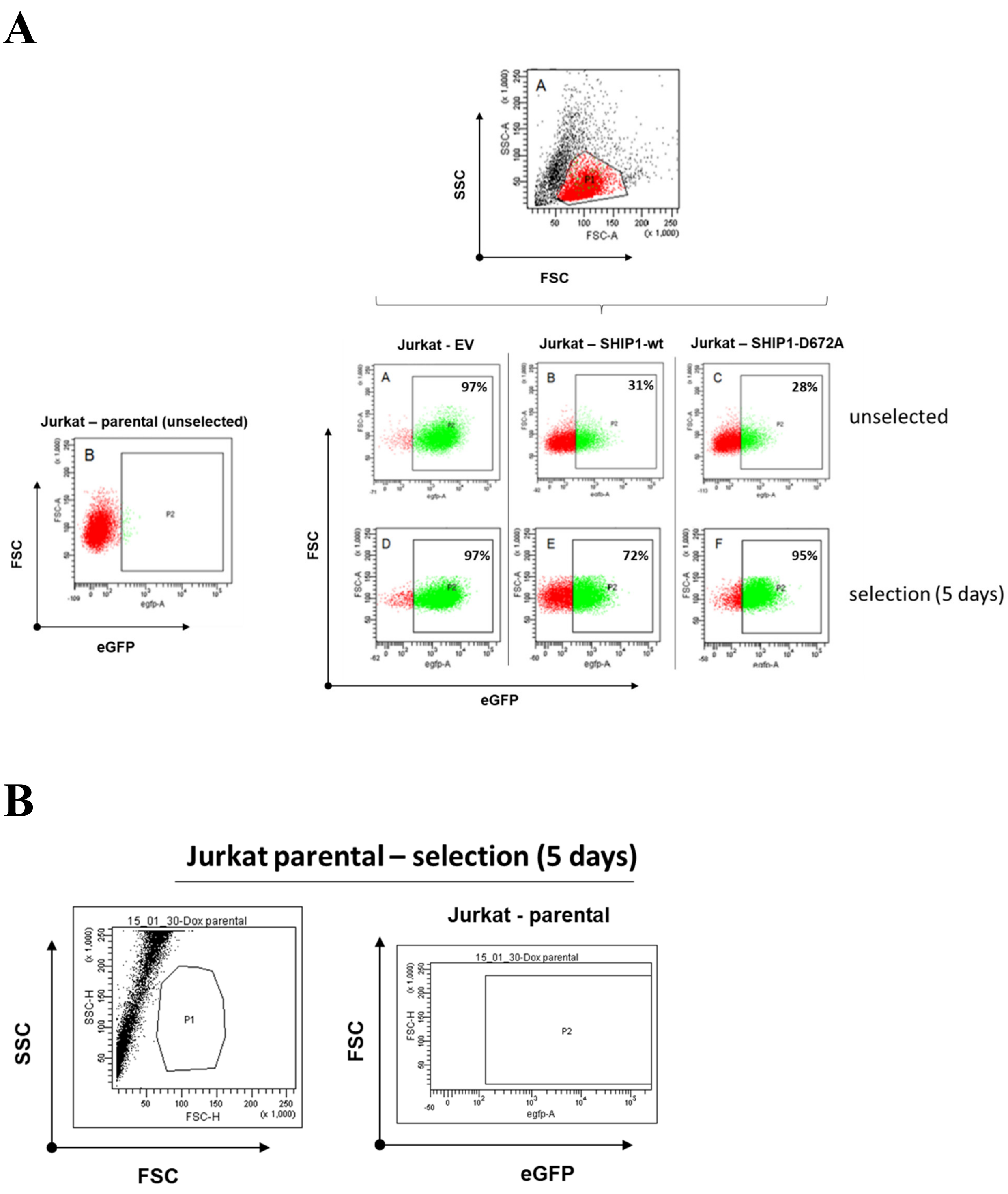

Figure S7: Representative flow cytometry gating strategy for analysis of eGFP positive Jurkat cell populations after puromycin selection. (A) The intact lymphocyte gate was analyzed depending on distinguished FSC vs. SSC properties. Vector transduced cells were identified by eGFP expression. To indicate the boundaries between eGFP negative and positive populations, gates were set according to parental Jurkat populations (without transduction). Five days after puromycin selection, the eGFP-positive cells were examined by flow cytometry compared to unselected cells. The percentage of eGFP-expressing cells is shown before and after treatment. (B) Flow cytometry analysis of puromycin selection of parental Jurkat cells. Intact lymphocytes in the lymphocyte gate depending on distinguished FSC vs. SSC properties were not found.

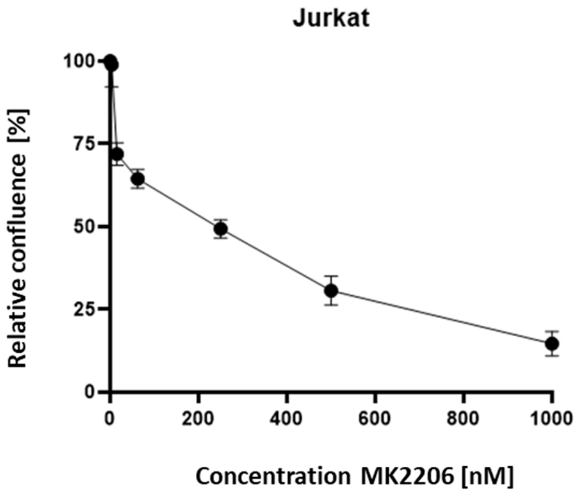

Figure S8: IC50 determination of MK2206 in Jurkat T-ALL cells. Jurkat cells were seeded in a cell density of 3 x 10^4 cells/well in a 96-well plate with 200 µl medium and placed for 48 hours in a live cell imaging system (IncuCyte ZOOM). For IC50 value determination cells were treated with various concentrations (1000, 500, 250, 62.5, 31.3, 3.9 nM) of MK2206. DMSO (solvent) was used as a control.

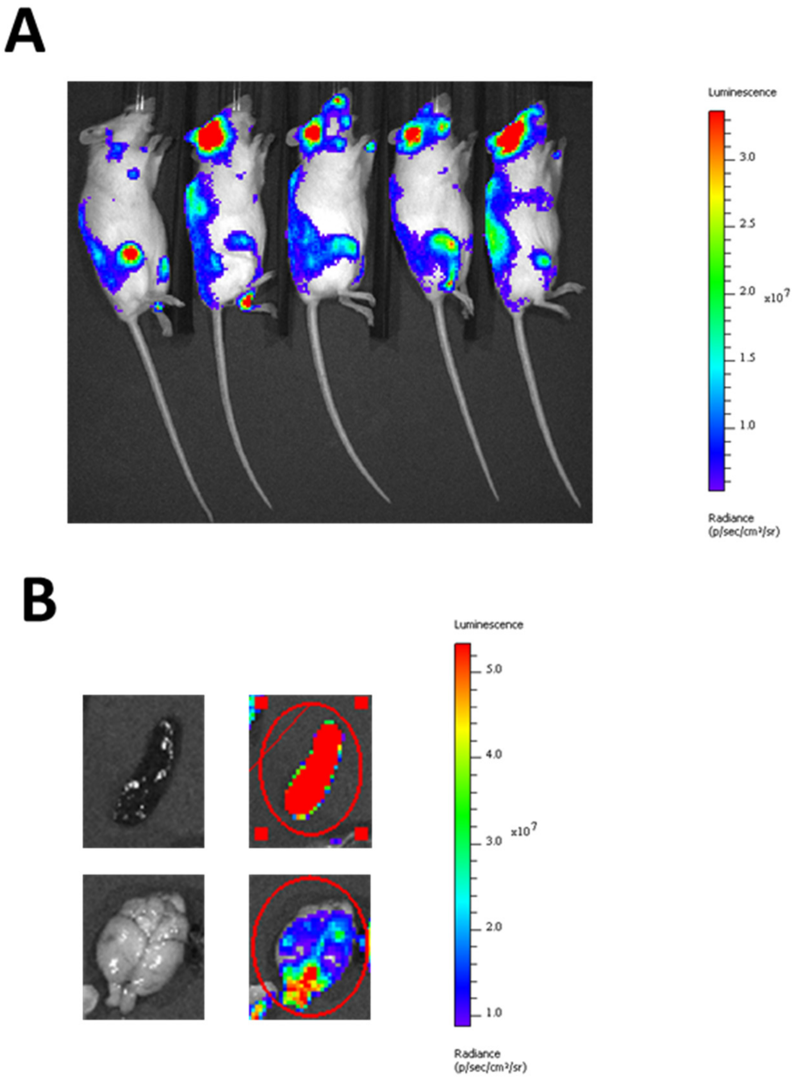

Figure S9: Jurkat cells aggressively invade the central nervous system after application in a murine xenograft model for preliminary investigation of the behaviour of T-ALL disease. (A) Representative IVIS images of mice that developed T-ALL disease with T-ALL cells in the central nervous system (brain and along the spinal cord) after three weeks of injection with Jurkat cells. The cells were tagged with a luciferase reporter gene. (B) Shown above is a representative image of an isolated spleen before and after IVIS analysis. Shown below is a representative image of the isolated brain before and after IVIS analysis. IVIS: In vivo imaging system.

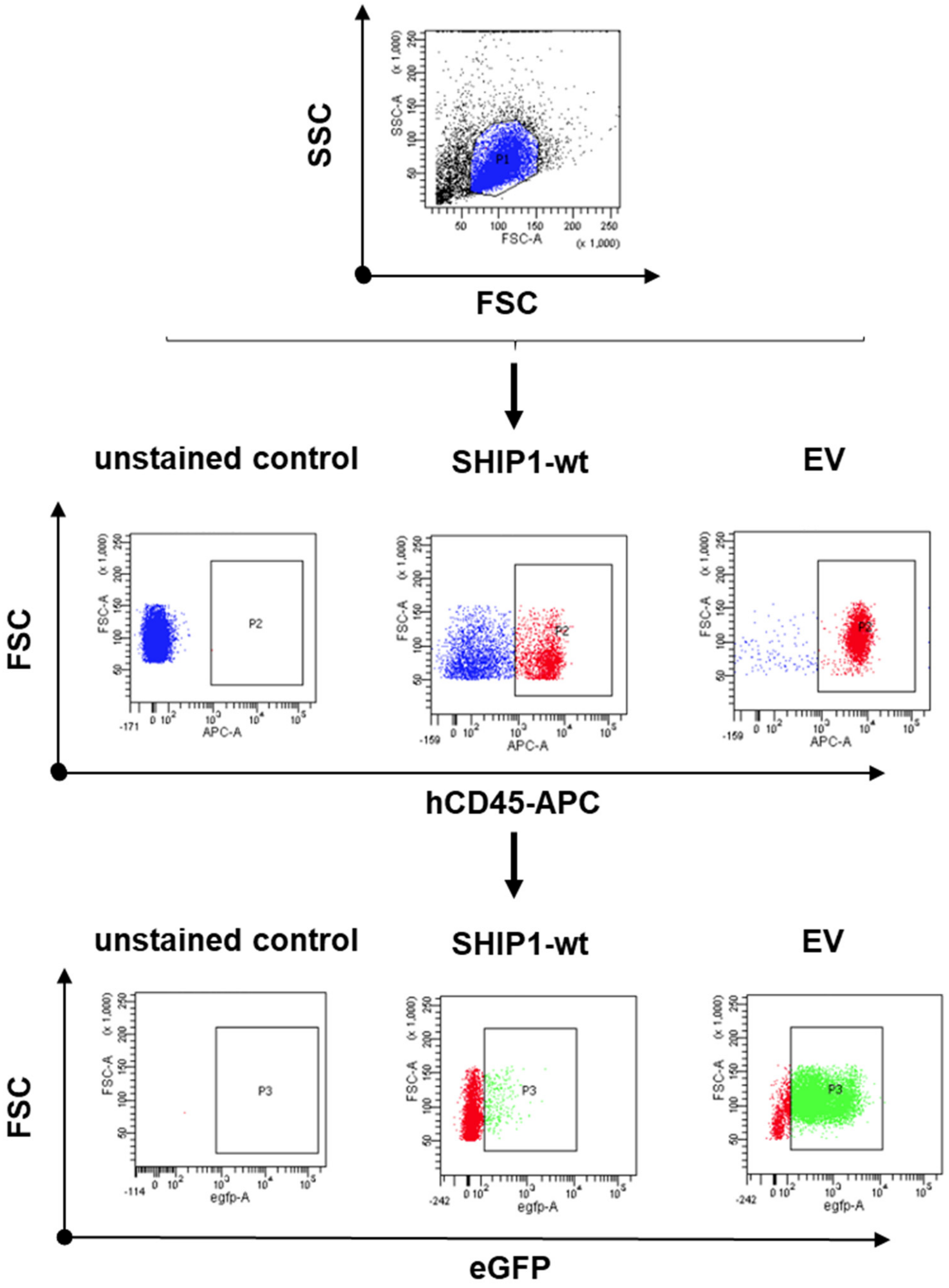

Figure S10: Representative flow cytometry gating strategy for analysis of eGFP positive human Jurkat cell populations after isolation of the bone marrow of xeno-transplantated mice. Diseased mice of the SHIP1-wt and control cohort were killed, the bone marrow was extracted from the hind bones and the cells were stained with an APC-coupled anti-hCD45 antibody in order to be able to identify the human Jurkat cells. The intact lymphocyte gate was analyzed depending on distinguished FSC vs. SSC properties. Vector transduced cells were identified by eGFP expression.

A

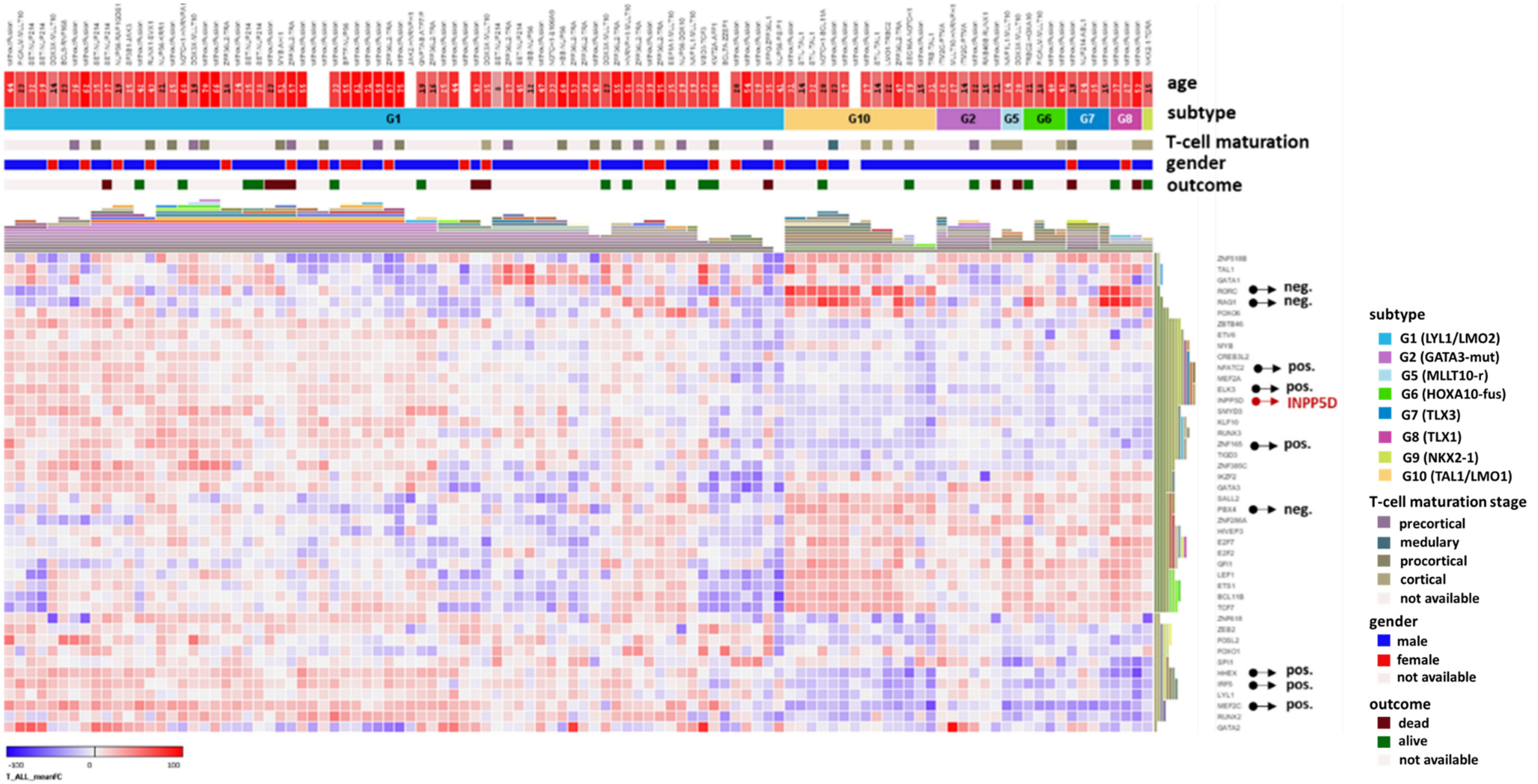

age
subtype
T-cell maturation
gender
outcome
G1
G10
G2
G5
G6
G7
G8
neg.
neg.
pos.
pos.
INPP5D
pos.
neg.
pos.
pos.
pos.
subtype
G1 (LYL1/LMO2)
G2 (GATA3-mut)
G5 (MLLT10-r)
G6 (HOXA10-fus)
G7 (TLX3)
G8 (TLX1)
G9 (NKX2-1)
G10 (TAL1/LMO1)
T-cell maturation stage
precortical
medulary
procortical
cortical
not available
gender
male
female
not available
outcome
dead
alive
not available

B

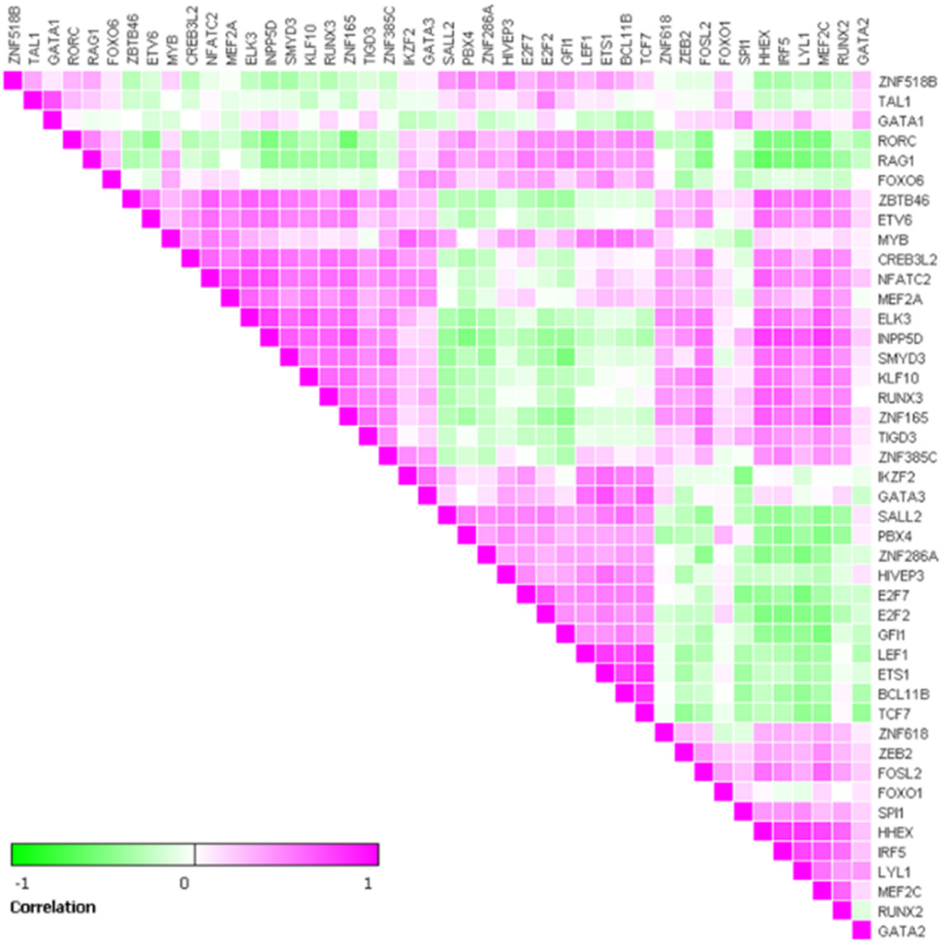
ZNF518B
TAL1
GATA1
RORC
RAG1
FOXO6
ZBTB46
ETV6
MYB
CREB3L2
NFATC2
MEF2A
ELK3
INPP5D
SMYD3
KLF10
RUNX3
ZNF165
TIGD3
ZNF385C
IKZF2
GATA3
SALL2
PBX4
ZNF286A
HIVEP3
E2F7
E2F2
GFI1
LEF1
ETS1
BCL11B
TCF7
ZNF618
ZEB2
FOSL2
FOXO1
SPI1
HHEX
IRF5
LYL1
MEF2C
RUNX2
GATA2
-1
0
1
Correlation

C

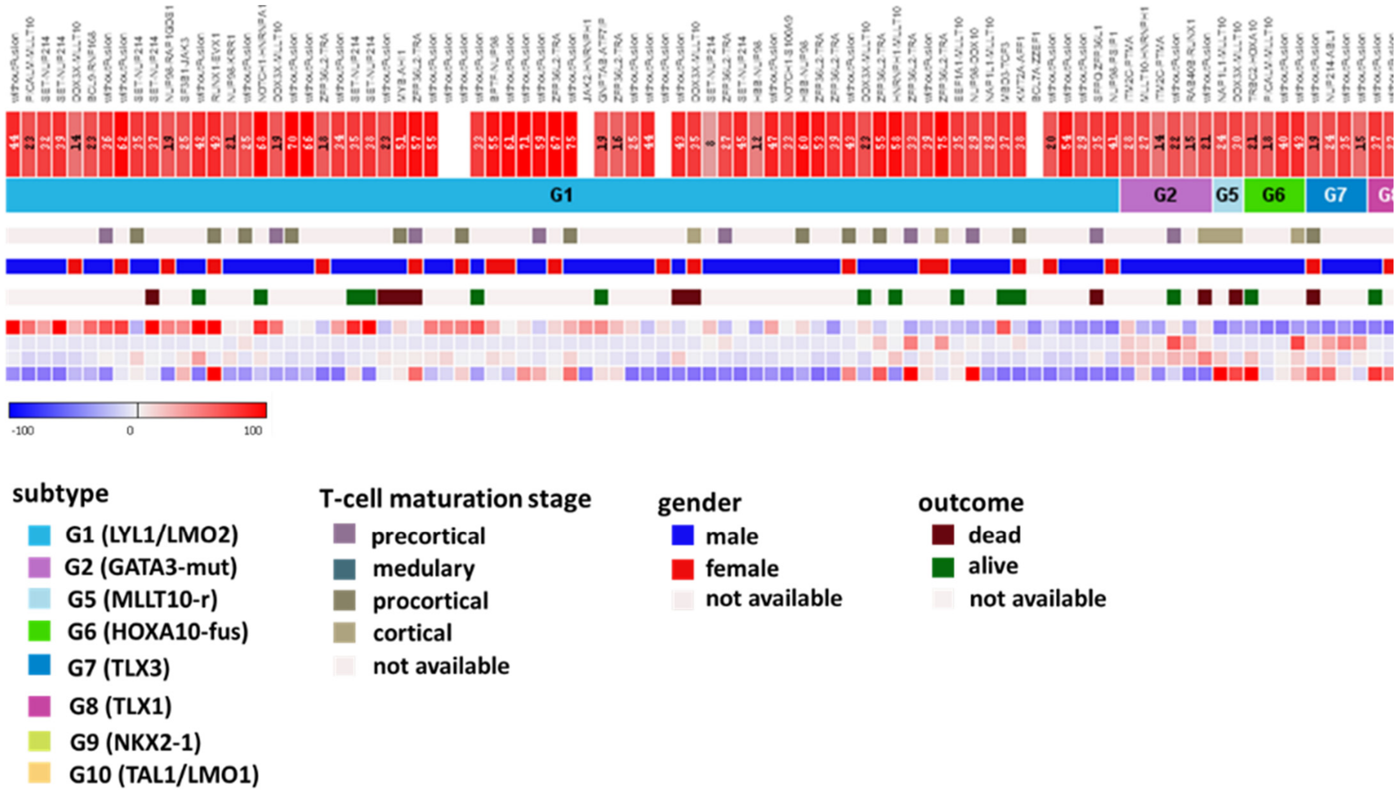

Figure S11: Gene expression analysis of transcription factors and RTKs according to molecular subtype in transcriptome data of 106 T-ALL patient samples. (A) Columns indicate T-ALL patients and clinical information (age, subtype, T-cell maturation stage, gender and clinical outcome), rows represent gene expression (mean fold change) of transcription factors after gene set enrichment analysis according to molecular subtype. Patient samples were arranged according to subgroup affiliation. For the gene-expression, up- and down-regulated genes are shown in the heatmap in red and blue, respectively. Ten subtypes are defined according to the molecular features defined by [36]: G1 (LYL1/LMO2 overexpression), G2 (GATA-3 mutation), G3 (SPI1-fusion), G4 (KMT2A-rearrangement), G5 (MLLT10-rearrangement), G6 (HOXA10-fusion), G7 (TLX3 overexpression), G8 (TLX1 overexpression), G9 (NKX2-1 overexpression) and G10 (TAL1/LMO1 overexpression). (B) Correlation analysis between the different genes of (A) was carried out. (C) Gene expression of INPP5D and selected RTKs by subgroup affiliation. Taken from the data set of [36]
